# Fungal and host transcriptome analysis of pH-regulated genes during colonization of apple fruits by *Penicillium expansum*

**DOI:** 10.1186/s12864-016-2665-7

**Published:** 2016-05-04

**Authors:** Shiri Barad, Noa Sela, Dilip Kumar, Amit Kumar-Dubey, Nofar Glam-Matana, Amir Sherman, Dov Prusky

**Affiliations:** Department of Postharvest Science of Fresh Produce, Agricultural Research Organization, the Volcani Center, Bet Dagan, 50250 Israel; Department of Plant Pathology and Microbiology, Robert H. Smith Faculty of Agriculture, Food and Environment, Hebrew University of Jerusalem, Rehovot, 76100 Israel; Department of Plant Pathology and Weed Research, ARO, the Volcani Center, Bet Dagan, 50250 Israel; Genomics Unit, ARO, the Volcani Center, Bet Dagan, 50250 Israel

**Keywords:** Transcription profiling, *Penicillium expansum*, RNA-seq, Gene expression, pH regulation, Pathogenicity, pH-regulated genes, Fungal genes regulated by pH, Apple genes regulated by pH

## Abstract

**Background:**

*Penicillium expansum* is a destructive phytopathogen that causes decay in deciduous fruits during postharvest handling and storage. During colonization the fungus secretes D-gluconic acid (GLA), which modulates environmental pH and regulates mycotoxin accumulation in colonized tissue. Till now no transcriptomic analysis has addressed the specific contribution of the pathogen's pH regulation to the *P. expansum* colonization process. For this purpose total RNA from the leading edge of *P. expansum*-colonized apple tissue of cv. 'Golden Delicious' and from fungal cultures grown under pH 4 or 7 were sequenced and their gene expression patterns were compared.

**Results:**

We present a large-scale analysis of the transcriptome data of *P. expansum* and apple response to fungal colonization. The fungal analysis revealed nine different clusters of gene expression patterns that were divided among three major groups in which the colonized tissue showed, respectively: (i) differing transcript expression patterns between mycelial growth at pH 4 and pH 7; (ii) similar transcript expression patterns of mycelial growth at pH 4; and (iii) similar transcript expression patterns of mycelial growth at pH 7. Each group was functionally characterized in order to decipher genes that are important for pH regulation and also for colonization of apple fruits by *Penicillium*. Furthermore, comparison of gene expression of healthy apple tissue with that of colonized tissue showed that differentially expressed genes revealed up-regulation of the jasmonic acid and mevalonate pathways, and also down-regulation of the glycogen and starch biosynthesis pathways.

**Conclusions:**

Overall, we identified important genes and functionalities of *P. expansum* that were controlled by the environmental pH. Differential expression patterns of genes belonging to the same gene family suggest that genes were selectively activated according to their optimal environmental conditions (pH, in vitro or in vivo) to enable the fungus to cope with varying conditions and to make optimal use of available enzymes. Comparison between the activation of the colonized host's gene responses by alkalizing *Colletotrichum gloeosporioides* and acidifying *P. expansum* pathogens indicated similar gene response patterns, but stronger responses to *P. expansum*, suggesting the importance of acidification by *P. expansum* as a factor in its increased aggressiveness.

**Electronic supplementary material:**

The online version of this article (doi:10.1186/s12864-016-2665-7) contains supplementary material, which is available to authorized users.

## Background

The genus *Penicillium* comprises a group of anamorphic fungi in the division Ascomycota [1]. Some *Penicillium* species are of economic importance because they are postharvest pathogens that cause spoilage in tropical and deciduous fruits. *Penicillium expansum* is the causal agent of blue mold, which is considered one of the most important postharvest pathogens [2] and that causes decay in deciduous fruits during postharvest handling and storage.

*Penicillium expansum* causes extensive maceration of the infected tissue by means of a common mechanism of tissue acidification [3]; it acidifies its host by activation of glucose oxidase 2 (gox2) and the catalyzed oxidation of glucose resulting in secretion of small molecules such as D-gluconic acid (GLA) [3, 4], which modulate the environmental pH and thereby activate several polygalacturonases that contribute to pectin depolymerization and consequently tissue maceration, at pH 3.5-4 [3, 5, 6]. Functional analysis of glucose oxidase 2-RNAi mutants showed a strong effect on pathogen interactions with their host: the greater the down-regulation of gox2, the stronger the impairment of GLA production, medium acidification, and apple fruit colonization [7]. More recently, Barad et al. [8] reported that *P. expansum* secreted not only GLA but also ammonia at the leading edge of colonization in apple fruits. Growth of the fungus in high-sucrose media induced rapid metabolism of sugar and increased GLA accumulation, and the decrease in available carbon reserves resulted in enhanced ammonia accumulation, probably because of amino acid catabolism, such as occurs with other pathogens [9, 10]. These results indicated that bidirectional pH regulation occurs in *P. expansum*-probably dependent on nutrient availability. The question concerns which metabolic processes are modulated by the pH level during colonization of apple fruits.

PacC is a transcription factor that activates genes of *Aspergillus nidulans* during the alkalization of the environment [11]. In a recent report [8], it was suggested that *P. expansum*'s *pacC* gene was active also under acidic conditions, and that ammonia produced at the leading edge of the decaying tissue under low-pH conditions further contributed to the activation of *pacC* responsiveness. This may indicate that *P. expansum* uses ammonia accumulation during nutritional modification of the ambient environmental pH as a regulatory cue for activation of *pacC*, by signaling and activating alkaline-induced genes that contribute to pathogenicity and accumulation of secondary metabolites such as patulin [8]. This pH modulation under the influence of GLA and ammonia accumulation is important for understanding the fungal response to differential gene regulation by pH; however, the overall response remains poorly understood.

The diversity of recently described ecological interactions of *Penicillium* with fruits [3, 4] and with other resident microorganisms [5] have suggested a wide variety of genes that may contribute to the pathogenicity of this pathogen. However, the work of Barad et al. [8] has attributed to the pH regulation of the host by *P. expansum* specific importance for pathogenicity. In the present study we used a transcriptomic approach to analyze the effect of pH on fungal gene regulation and host responses to various pH-modulating pathogens. The effect of the fungal response was observed by comparing the responses of the apple to colonization by *P. expansum* as driven by expression of the fungal gene at pH 4 or 7. Our RNA-Seq data of fungal responses revealed nine differentially co-expressed gene clusters; they showed alterations of expression patterns that were associated with differential pH responses, and also with genes related to *P. expansum* colonization. The apple response to pH was elucidated by using qRT-PCR to analyze expressions of selected genes in response to acidifying and alkalinizing pathogens: *P. expansum* and *Colletotrichum gloeosporioides,* respectively. This analysis indicated similar patterns of host response to the respective pathogens, but the apple genes showed an extensive multifold greater response to infection by its natural pathogen *P. expansum* than to that by *C. gloeosporioides.* This may indicate the importance of the acidifying modulation of pH by *Penicillium* as a factor for greater aggressiveness of *P. expansum*.

## Results and Discussion

### Profiling the expression of P. expansum genes during colonization and growth at pH 4 and 7

Genomes of *P. expansum* [7] were sequenced by using paired-end reads by means of Illumina Hiseq 2500 [12]. For analysis of the *P. expansum* transcriptome we downloaded the reference draft genome accession JQFX00000000.1 from the Genbank site [13]. Ten libraries of single-end RNAseq (deposited in Genbank under accession SRP071104) were mapped to the reference genome by using the Bowtie2 software [14]. The 10 libraries contained the following features: (i) *P. expansum* grown in culture media at pH 4 for 3 h as well as a pooled sample collected at several time points-0.5, 1, 3, 10, and 24 h-with two replicates at each point; (ii) *P. expansum* grown in culture media at pH 7 for 3 h, as well as a pool collected at several time points-0.5, 1, 3, 10, and 24 h-with two replicates at each point; and (iii) the leading edge, comprising 1–2 mm of apple cv. 'Golden Delicious' tissue, colonized by *P. expansum.* For in vitro experiments, 10^6^ spores of *P. expansum* were inoculated into germinating minimal medium (GMM) at pH 4.5 as described by Barad et al. [7] and transferred 2 days later to buffered inducing media at pH 4 or 7. At several different time points after transfer to inducing secondary media (SM), fungal mycelia were sampled and pooled for analysis. Pooled samples of RNA were compared with independent samples 3 h after transfer to inducing media.

**Fig. 1:**
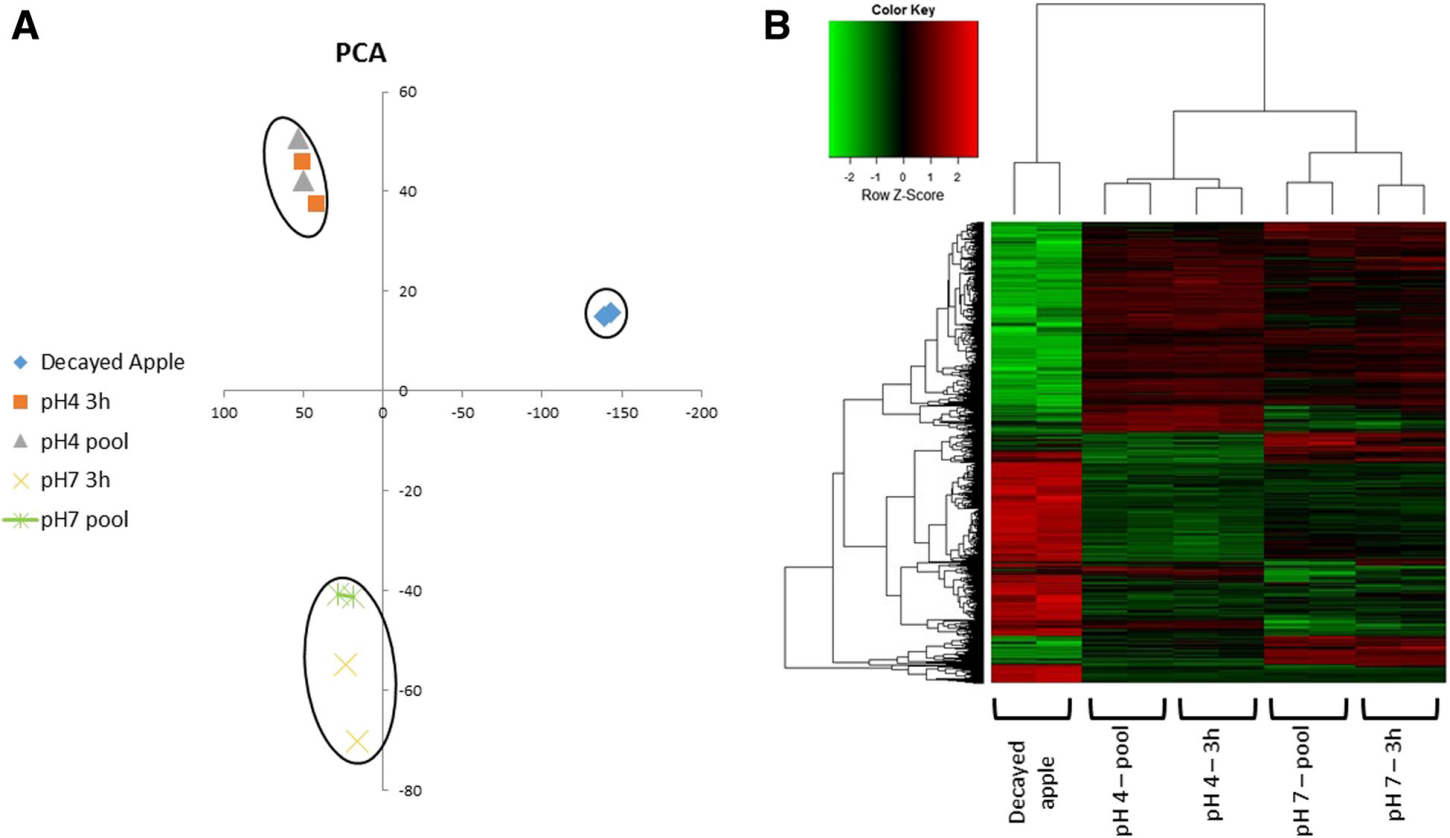
Principal component analysis (PCA) and heat map analysis of all the samples: duplicates of leading edge of inoculated apple; duplicates of pooled samples from all time points treated in vitro at pH 4.0; two replicates of *P. expansum* mycelia treated in vitro at pH 4.0 for 3 h; duplicates of a pool of samples from all time points treated in vitro at pH 7.0; and two replicates of *P. expansum* mycelia treated in vitro at pH 7.0 for 3 h. **a** PCA based on 5,646 differentially expressed genes. The results are plotted as: (♦) decayed apple; (■) in vitro at pH 4 for 3 h; (▲) pooled samples at pH 4; (X) in vitro at pH 7 for 3 h; (ӿ) pooled samples at pH 7. Circles are drawn around clustered points. **b** Expression heat map of differentially expressed *P. expansum* genes in the various samples

Principal component analysis (PCA) of all expressed genes of *P. expansum* indicated that the replicates were very close to each other, and that the expression patterns of fungal genes exposed to the respective pH conditions for 3 h were almost identical to those of pooled samples of mycelia exposed for various intervals (Fig. 1a). Moreover, the general pattern of gene expression in decayed apple tissue was far different from those of cultures grown under different pH conditions, as could be expected (Fig. 1a). Hierarchical clustering analysis indicated differing patterns of gene expression between the in vivo and in vitro samples at both pH levels; and the colonized apple tissue contained more down- than up-regulated genes (Fig. 1b).

Out of 11,019 annotated genes [12], 5,646 genes were differentially expressed according to an false discovery rate (FDR, [15]) threshold < 0.05 and the log fold change was greater than 1 or smaller than −1 implying at least 2 fold change in expression scale (Fig. 1b). We sorted the differentially expressed gene patterns into nine different co-expressed gene clusters, which fell into three major groups:

Group I comprised clusters of genes whose expression patterns in the colonized tissue differed with respect to growth at pH 4 or pH 7. This group comprised clusters 1, 2, 3, and 6, which contained genes that are important for *P. expansum* colonization within apple fruits.

Group II comprised clusters in which expression patterns of the colonized tissue were similar to those of the pathogen's growth in culture at pH 4, and differed from those of fungal growth at pH 7. This group includes clusters numbers 5, 7, and 8.

**Fig. 2:**
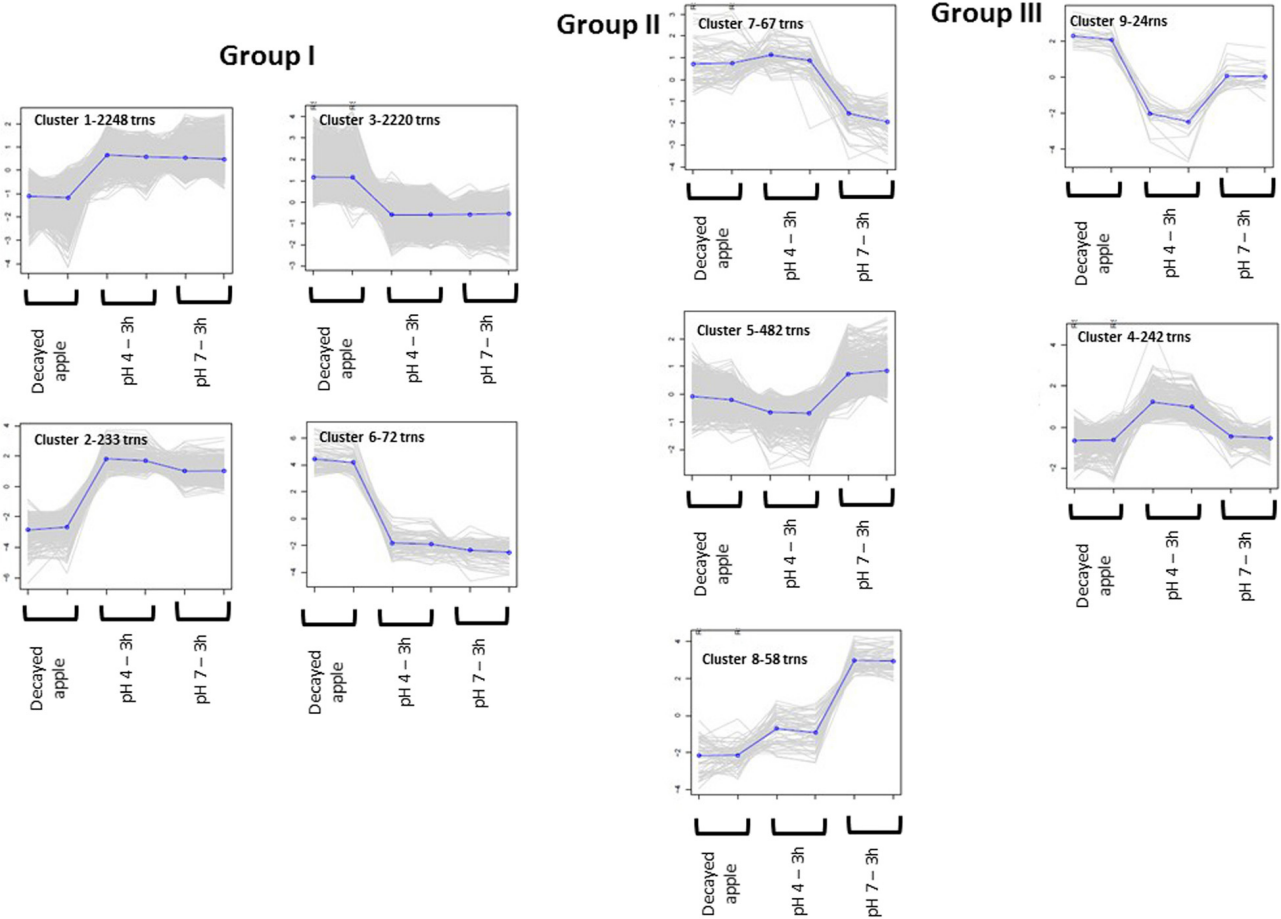
Expression patterns of 9 clusters of co-expressed differential genes. The normalized expression pattern (log 2-transformed, median centered) of 9 clusters that were derived from the hierarchical clustering algorithm by use of the hclust function algorithm in R [70, 71]. Each gene is plotted in gray, in addition to the mean expression profile for that cluster (blue). The figure was generated by using perl script define_clusters_by_cutting_tree.pl of the Trinity software package [67]

Group III comprised gene clusters in which expression patterns of the colonized tissue were similar to those expressed by the pathogen grown in culture at pH 7. This group includes clusters numbers 4 and 9 (Fig. 2).

Group I includes clusters of expressed genes, whose level of expression under in vitro conditions differed from that exhibited during colonization. This group comprised clusters 1 and 2, with 2,248 and 233 genes, respectively, which exhibited higher expression under in vitro growth conditions at pH 4 or 7 than that manifested as down-regulation in the colonized apple tissue (Fig. 2). The genes expressed under both high and low pH are probably important for growth under in vitro conditions but not for pathogenicity. Group I also included clusters 3 and 6, with 2,220 and 72 genes, respectively, which comprise genes that are up-regulated in apple tissue colonization but down-regulated in vitro at both at pH 4 and pH 7. All the genes in this group probably are involved in growth in either nutritional environments or liquids, or both, that differ substantially from those within the apple tissue.

Group II comprised clusters 5, 7, and 8, in which the gene expression patterns in the colonized tissue were similar to those in vitro at pH 4 but different from those in vitro at pH 7 (Fig. 2). Cluster 7, with a gene count of 67 transcripts, showed similar overall expression levels during colonization of apple tissue and during in vitro growth at pH 4, which suggests that this cluster contained genes that are activated at pH 4, i.e., the usual fruit pH, therefore we consider that they may contribute to colonization at pH 4. Another two clusters present in this group were 5 and 8, with gene counts of 482 and 58, respectively; they include genes that were down-regulated both in colonized apple tissue and in vitro at pH 4, but maintained high expression levels in vitro at pH 7. These genes are probably necessary for growth under alkaline conditions.

Group III comprises gene clusters that showed similar expression levels in colonized apple tissue and during in vitro growth fungal at pH 7, but showed altered gene expression at pH 4 (Fig. 2). This group included clusters 9 and 4, with 24 and 242 transcript counts, respectively. Genes in cluster 9 showed similar high expression levels in colonized fruit and in vitro at pH 7, but low levels in vitro at pH 4; these genes probably are expressed in fruits under induced alkaline conditions or during fungal ammonia accumulation [8]. Cluster 4 included genes that exhibited low expression levels both in colonized fruit and in vitro at pH 7; we hypothesize that these genes are repressed under alkaline conditions or during ammonia secretion [8].

### Gene ontology analysis of decayed apple tissue compared with in vitro conditions

We analyzed functional enrichment in each cluster using blast2go software [16] and Fisher’s Exact Test [17].

### Clusters with different gene expression levels under colonization than under in vitro conditions

#### Higher expression levels of fungal genes when grown in culture than in colonized tissue

Genes in clusters 1 and 2 showed higher expression levels under in vitro conditions than in colonized tissue. Functionality of these genes showed involvement in respiratory activity, in light of their easier growth in culture media than in colonized apple tissue. GO-enriched terms in clusters 1 and 2 are depicted in Additional file 1: Figure S1 and Additional file 2: Figure S2, respectively.

#### Lower expression levels of fungal genes when grown in culture than in colonized tissue

Genes in clusters 3 and 6 showed lower expression levels when grown in vitro at pH 4 or pH 7 than when grown in colonized tissue (Fig. 2). These clusters included genes encoding for vesicle transport, which might indicate activation of secretion process(es); probably of mycotoxin(s) and effectors (Additional file 3: Figure S3 and Additional file 4: Figure S6 respectively). Cluster 3 included most of the 15 genes involved in patulin biosynthesis-patB, D, E, F, H, I, J, K, L, and O. Unexpectedly, these genes were not highly expressed in vitro at either pH 4 or pH 7, which supports a previous report by Li et al. [18], which suggested the importance of specific nutritional growth conditions for induced expression of the patulin biosynthesis gene cluster (PatA-PatO) in *P. expansum*. However, at the same time, the question arises of the specific mechanism(s)-possibly nutritional and possibly others-that modulate the conditions for expression of the mycotoxin cluster in vivo [18–20]. Cluster 3 also showed genes such as acetamidase, amidase-family proteins, and fatty-acid amide hydrolase, encoding for amidase activity (Additional file 3: Figure S3) these may indicate a possible source of NH_4_^+^ production during periods of limited sucrose levels and pathogenicity of *P. expansum*, which would support earlier suggestions by Barad et al. [8] that local pH modulation might result from ammonia accumulation in the leading edge during pathogenicity of *P. expansum*.

Cluster 6 shows enriched expression of genes associated with host-cell-wall degradation that are important for the virulence of *P. expansum* [5] (Additional file 4: Figure. S6). These genes have previously known functionalities associated with pathogenicity of fungi; these genes and activities include: chitinase-associated genes, pectin lyase, and polygalacturonase (PG) activities. Among these, PG was shown to be involved in maceration of apples by *P. expansum*, particularly in diseases characterized by tissue maceration or soft rot [21]; and pectin lyases contribute to the pathogenicity of many pathogens by degrading pectin polymers directly by means of a β-elimination mechanism that results in formation of 4,5-unsaturated oligo-galacturonides [22]. In addition, cluster 6 shows over-representation of genes encoding for aspartic endopeptidase-pep1-which is associated with pH modulation and pathogenicity of *P. digitatum* [23]. Aspartic endopeptidase catalyzes hydrolysis of elastin and collagen, the major structural proteins of basement membranes, and plays a significant role in virulence of *P. digitatum* on citrus fruits [23]. Aspartic endopeptidase was up-regulated during infection of citrus fruits, and contributed to fungal colonization, either by degradation of plant cell-wall components to provide a nitrogen supply, or even by inactivating defense proteins [24]. This type of response at low pH is probably a result of accumulation of GLA during the pathogenicity process of *P. expansum*, in order to ensure that secreted enzymes and metabolites are produced at the optimal pH to fully facilitate their physiological functions [3].

### Clusters with similar expression levels in colonized apple and in vitro at pH 4

#### High expression level of fungal genes in colonized apple tissue and in samples grown in culture at pH 4

A single cluster-cluster 7-showed enhanced expression of genes both in colonized apple tissue and in vitro at pH 4 (Fig. 2). This cluster was enriched in genes involved in the glutamate metabolic process (Additional file 5: Figure S7); a process that includes high expression of glutamate decarboxylase (Table 1), which is required for normal oxidative-stress tolerance in *Saccharomyces cerevisiae*. γ-Aminobutyric acid (GABA) largely originates from decarboxylation of L-glutamate; it is associated with sporulation/spore metabolism [25] and in most fungi it serves as a carbon and nitrogen source. Also, γ-aminobutyric acid metabolism promotes asexual sporulation in *Saccharomyces nodorum* [26]. Moreover, in plants and fungi GABA synthesis has been associated with acidic pH, either in response to cytosolic acidification-probably as a pH-regulatory mechanism-or during growth under acidic conditions [25]. Since acidification of the host environment usually occurs during apple colonization by *P. expansum*, it is possible that γ-aminobutyric acid synthesis might be one of the mechanisms employed by the fungus to cope with this more acidic pH of the environment [6].

**Table 1:** Genes involved in each process of cluster 7

GO Term	Gene id	Genbank transcript id	Description
Nucleobase transport	PEXP_081890	KGO47390	Permease, cytosine/purine, uracil, thiamine, allantoin
	PEXP_004400	KGO37467	Permease, cytosine/purine, uracil, thiamine, allantoin
	PEXP_081870	KGO47388	Permease, cytosine/purine, uracil, thiamine, allantoin
Taurine metabolic process	PEXP_099510	KGO46394	Pyridoxal phosphate-dependent transferase, major region, subdomain 1
	PEXP_039450	KGO48081	Pyridoxal phosphate-dependent transferase, major region, subdomain 1
Beta-alanine metabolic process	PEXP_099510	KGO46394	Pyridoxal phosphate-dependent transferase, major region, subdomain 1
	PEXP_039450	KGO48081	Pyridoxal phosphate-dependent transferase, major region, subdomain 1
Transmembrane transport	PEXP_023020	KGO42773	Major facilitator superfamily domain, general substrate transporter
	PEXP_104460	KGO36460	Major facilitator superfamily domain, general substrate transporter
	PEXP_019050	KGO45782	C4-dicarboxylate transporter/malic acid transport protein
	PEXP_083120	KGO38619	Major facilitator superfamily domain, general substrate transporter
	PEXP_105230	KGO41447	Major facilitator superfamily domain, general substrate transporter
	PEXP_012370	KGO49188	Major facilitator superfamily domain, general substrate transporter
	PEXP_060340	KGO45245	Iron permease FTR1
	PEXP_059590	KGO45345	Major facilitator superfamily domain, general substrate transporter
	PEXP_094700	KGO43568	Major facilitator superfamily domain, general substrate transporter
	PEXP_025230	KGO42652	Major facilitator superfamily domain, general substrate transporter
	PEXP_030850	KGO40270	ABC transporter, integral membrane type 1
	PEXP_078840	KGO37868	Major facilitator superfamily domain, general substrate transporter
Glutamate metabolic process	PEXP_099510	KGO46394	Pyridoxal phosphate-dependent transferase, major region, subdomain 1
	PEXP_039450	KGO48081	Pyridoxal phosphate-dependent transferase, major region, subdomain 1
Glutamate decarboxylase activity	PEXP_099510	KGO46394	Pyridoxal phosphate-dependent transferase, major region, subdomain 1
	PEXP_039450	KGO48081	Pyridoxal phosphate-dependent transferase, major region, subdomain 1
Nucleobase transmembrane transporter activity	PEXP_081890	KGO47390	Permease, cytosine/purine, uracil, thiamine, allantoin
	PEXP_004400	KGO37467	Permease, cytosine/purine, uracil, thiamine, allantoin
	PEXP_081870	KGO47388	Permease, cytosine/purine, uracil, thiamine, allantoin
Cofactor binding	PEXP_107000	KGO41320	Aldolase-type TIM barrel
	PEXP_068570	KGO46687	Aldolase-type TIM barrel
	PEXP_099510	KGO46394	Pyridoxal phosphate-dependent transferase, major region, subdomain 1
	PEXP_080260	KGO38010	Pyridoxal phosphate-dependent transferase, major region, subdomain 2
	PEXP_043260	KGO39485	Thiamine pyrophosphate enzyme, C-terminal TPP-binding
	PEXP_076130	KGO36094	D-isomer specific 2-hydroxyacid dehydrogenase, NAD-binding
	PEXP_001130	KGO44422	Glyceraldehyde/Erythrose phosphate dehydrogenase family
	PEXP_039450	KGO48081	Pyridoxal phosphate-dependent transferase, major region, subdomain 1
Glucosylceramidase activity	PEXP_081260	KGO47327	Glycoside hydrolase, family 30
3-isopropylmalate dehydratase activity	PEXP_074470	KGO48717	Aconitase/3-isopropylmalate dehydratase large subunit, alpha/beta/alpha, subdomain 1/3
N-acylphosphatidylethanolamine-specific phospholipase D activity	PEXP_024950	KGO42624	N-acyl-phosphatidylethanolamine-hydrolysing phospholipase D
Transmembrane transporter activity	PEXP_104460	KGO36460	Major facilitator superfamily domain, general substrate transporter
	PEXP_034210	KGO40038	CDR ABC transporter
	PEXP_019050	KGO45782	C4-dicarboxylate transporter/malic acid transport protein
	PEXP_105230	KGO41447	Major facilitator superfamily domain, general substrate transporter
	PEXP_081890	KGO47390	Permease, cytosine/purine, uracil, thiamine, allantoin
	PEXP_004400	KGO37467	Permease, cytosine/purine, uracil, thiamine, allantoin
	PEXP_030850	KGO40270	ABC transporter, integral membrane type 1
	PEXP_081870	KGO47388	Permease, cytosine/purine, uracil, thiamine, allantoin
Integral component of membrane	PEXP_023020	KGO42773	Major facilitator superfamily domain, general substrate transporter
	PEXP_104460	KGO36460	Major facilitator superfamily domain, general substrate transporter
	PEXP_034210	KGO40038	CDR ABC transporter
	PEXP_019050	KGO45782	C4-dicarboxylate transporter/malic acid transport protein
	PEXP_083120	KGO38619	Major facilitator superfamily domain, general substrate transporter
	PEXP_054930	KGO44009	Major intrinsic protein
	PEXP_105230	KGO41447	Major facilitator superfamily domain, general substrate transporter
	PEXP_012370	KGO49188	Major facilitator superfamily domain, general substrate transporter
	PEXP_059590	KGO45345	Major facilitator superfamily domain, general substrate transporter
	PEXP_094700	KGO43568	Major facilitator superfamily domain, general substrate transporter
	PEXP_027630	KGO43014	Amino acid transporter, transmembrane
	PEXP_025230	KGO42652	Major facilitator superfamily domain, general substrate transporter
	PEXP_030850	KGO40270	ABC transporter, integral membrane type 1
	PEXP_038200	KGO47945	Mitochondrial substrate/solute carrier
	PEXP_078840	KGO37868	Major facilitator superfamily domain, general substrate transporter
Lysosome	PEXP_081260	KGO47327	Glycoside hydrolase, family 30
3-Isopropylmalate dehydratase complex	PEXP_074470	KGO48717	Aconitase/3-isopropylmalate dehydratase large subunit, alpha/beta/alpha, subdomain 1/3

#### Low expression level of fungal genes in colonized apple and in samples grown in vitro at pH 4

In contrast to the single up-regulated cluster 7, two clusters-5 and 8-showed repressed expression of genes both in colonized apple tissue and in vitro at pH 4 (Fig. 2). Cluster 5 showed enrichment of genes that are involved in fungal pathogenicity and that are known to be active under alkaline conditions; they exhibit, for example, pectate lyase and hydrolase activities [27] (Additional file 6: Figure S5) and the transcription factor PacC (Table 2), which suggests that the acidic pH-repressed alkaline-expressed genes are involved in pathogenicity. It is likely that *P. expansum* adopted its acidifying life pattern characterized by production of organic acids-mainly gluconic acid secretion [7]-in order to optimize the pH of the media. These present findings support a previous publication by Barad et al. [28] which indicated that whereas *pacC* expression was inhibited under acidic conditions during the accumulation of gluconic acid, it might be activated in the leading edge of colonized tissue where ammonia is accumulated at pH 4 [8]. Part of the localized pH modulation may be regulated by the Na^+^ and Ca^2+^ transporters that are present in cluster 5, that modulate the internal equilibrium of hydrogen ions and contribute to cytoplasmic pH (Table 2).

**Table 2:** Genes involved in each process of cluster 5

GO Term	Gene id	Genbank transcript id	Description
Regulation of pH	PEXP_046990	KGO39741	Sodium/calcium exchanger membrane region
	PEXP_043720	KGO39291	Alkali metal cation/H+ antiporter Nha1, C-terminal
	PEXP_009670	KGO49300	Cation/H+ exchanger
	PEXP_095400	KGO43638	Sodium/calcium exchanger membrane region
L-Arabinose metabolic process	PEXP_023030	KGO42774	Glycoside hydrolase, superfamily
	PEXP_071150	KGO40611	Alcohol dehydrogenase superfamily, zinc-type
	PEXP_089320	KGO41720	Glycoside hydrolase, superfamily
Calcium ion transport	PEXP_046990	KGO39741	Sodium/calcium exchanger membrane region
	PEXP_088420	KGO41618	ATPase, P-type, K/Mg/Cd/Cu/Zn/Na/Ca/Na/H-transporter
	PEXP_095400	KGO43638	Sodium/calcium exchanger membrane region
	PEXP_050270	KGO42077	ATPase, P-type, K/Mg/Cd/Cu/Zn/Na/Ca/Na/H-transporter
Kynurenine metabolic process	PEXP_039010	KGO48037	putative cyclase
	PEXP_045070	KGO39237	putative cyclase
	PEXP_009350	KGO49268	Pyridoxal phosphate-dependent transferase, major region, subdomain 2
Glyoxylate cycle	PEXP_011610	KGO49112	Malate synthase A
	PEXP_077890	KGO37773	Pyruvate/Phosphoenolpyruvate kinase
Tryptophan catabolic process	PEXP_039010	KGO48037	Putative cyclase
	PEXP_045070	KGO39237	Putative cyclase
	PEXP_009350	KGO49268	Pyridoxal phosphate-dependent transferase, major region, subdomain 2
Inorganic ion transmembrane transport	PEXP_059490	KGO45335	Sulfate anion transporter
	PEXP_043720	KGO39291	Alkali metal cation/H+ antiporter Nha1, C-terminal
	PEXP_048620	KGO39686	Nickel/cobalt transporter, high-affinity
	PEXP_009670	KGO49300	Cation/H+ exchanger
	PEXP_088420	KGO41618	ATPase, P-type, K/Mg/Cd/Cu/Zn/Na/Ca/Na/H-transporter
	PEXP_095400	KGO43638	Sodium/calcium exchanger membrane region
	PEXP_050270	KGO42077	ATPase, P-type, K/Mg/Cd/Cu/Zn/Na/Ca/Na/H-transporter
	PEXP_094650	KGO43563	Ammonium transporter
Disaccharide metabolic process	PEXP_052570	KGO42305	Glycosyl transferase, family 20
	PEXP_011870	KGO49138	Pectinesterase, catalytic
	PEXP_069330	KGO46763	Glycoside hydrolase, family 61
	PEXP_011880	KGO49139	Glycoside hydrolase, family 28
	PEXP_066270	KGO46936	Hexokinase, N-terminal
	PEXP_053240	KGO42372	Glycoside hydrolase, family 61
	PEXP_107940	KGO41788	Alpha-amylase, C-terminal all beta
Substrate-specific transmembrane transporter activity	PEXP_046990	KGO39741	Sodium/calcium exchanger membrane region
	PEXP_059490	KGO45335	Sulfate anion transporter
	PEXP_016910	KGO46049	Xanthine/uracil/vitamin C permease
	PEXP_043720	KGO39291	Alkali metal cation/H+ antiporter Nha1, C-terminal
	PEXP_052330	KGO42281	Major facilitator superfamily domain, general substrate transporter
	PEXP_033600	KGO39977	Amino acid/polyamine transporter I
	PEXP_094860	KGO43584	Major facilitator superfamily domain, general substrate transporter
	PEXP_068270	KGO46657	Nucleobase cation symporter-1, NCS1
	PEXP_006100	KGO36811	Major facilitator superfamily domain, general substrate transporter
	PEXP_086200	KGO40808	Amino acid/polyamine transporter I
	PEXP_003610	KGO37353	Major facilitator superfamily domain, general substrate transporter
	PEXP_043410	KGO39260	Major facilitator superfamily domain, general substrate transporter
	PEXP_048620	KGO39686	Nickel/cobalt transporter, high-affinity
	PEXP_006360	KGO36837	Major facilitator superfamily domain, general substrate transporter
	PEXP_000260	KGO44335	Amino acid/polyamine transporter I
	PEXP_072400	KGO48510	Major facilitator superfamily domain, general substrate transporter
	PEXP_009030	KGO48810	Na dependent nucleoside transporter
	PEXP_068910	KGO46721	Major facilitator superfamily domain, general substrate transporter
	PEXP_031270	KGO40312	Permease, cytosine/purine, uracil, thiamine, allantoin
	PEXP_009670	KGO49300	Cation/H+ exchanger
	PEXP_110420	KGO38764	ATPase, P-type, K/Mg/Cd/Cu/Zn/Na/Ca/Na/H-transporter
	PEXP_095790	KGO43677	Major facilitator superfamily domain, general substrate transporter
	PEXP_048590	KGO39683	Amino acid/polyamine transporter I
	PEXP_104220	KGO36561	Cation/H+ exchanger
	PEXP_019490	KGO45826	C4-dicarboxylate transporter/malic acid transport protein
	PEXP_088420	KGO41618	ATPase, P-type, K/Mg/Cd/Cu/Zn/Na/Ca/Na/H-transporter
	PEXP_076320	KGO37552	Major facilitator superfamily domain, general substrate transporter
	PEXP_001370	KGO37112	Major facilitator superfamily domain, general substrate transporter
	PEXP_084160	KGO41053	Amino acid/polyamine transporter I
	PEXP_095400	KGO43638	Sodium/calcium exchanger membrane region
	PEXP_050270	KGO42077	ATPase, P-type, K/Mg/Cd/Cu/Zn/Na/Ca/Na/H-transporter
	PEXP_062420	KGO45644	General substrate transporter
	PEXP_022750	KGO42746	Major facilitator superfamily domain, general substrate transporter
	PEXP_068690	KGO46699	Major facilitator superfamily domain, general substrate transporter
	PEXP_034320	KGO40048	Arsenical pump membrane protein, ArsB
	PEXP_074940	KGO36159	Major facilitator superfamily domain, general substrate transporter
	PEXP_094650	KGO43563	Ammonium transporter
Solute:proton antiporter activity	PEXP_046990	KGO39741	Sodium/calcium exchanger membrane region
	PEXP_043720	KGO39291	Alkali metal cation/H+ antiporter Nha1, C-terminal
	PEXP_009670	KGO49300	Cation/H+ exchanger
	PEXP_104220	KGO36561	Cation/H+ exchanger
	PEXP_095400	KGO43638	Sodium/calcium exchanger membrane region
Cation:cation antiporter activity	PEXP_046990	KGO39741	Sodium/calcium exchanger membrane region
	PEXP_043720	KGO39291	Alkali metal cation/H+ antiporter Nha1, C-terminal
	PEXP_104220	KGO36561	Cation/H+ exchanger
	PEXP_095400	KGO43638	Sodium/calcium exchanger membrane region
Metal ion binding	PEXP_046510	KGO39177	Cytochrome P450, E-class, CYP52
	PEXP_036890	KGO35901	Transcription factor, fungi
	PEXP_084560	KGO41093	4-Hydroxyphenylpyruvate dioxygenase
	PEXP_055320	KGO44077	Polyketide synthase, enoylreductase
	PEXP_042410	KGO39400	Manganese/iron superoxide dismutase, C-terminal
	PEXP_072700	KGO48540	Annexin
	PEXP_089160	KGO41704	Terpenoid synthase
	PEXP_102680	KGO36423	Transcription factor, fungi
	PEXP_042010	KGO48399	Protein of unknown function DUF3468
	PEXP_011320	KGO49083	Zinc finger, C2H2
	PEXP_095680	KGO43666	Protein of unknown function DUF3468
	PEXP_050580	KGO42108	Cytochrome P450
	PEXP_059140	KGO45293	Polyketide synthase, enoylreductase
	PEXP_100050	KGO46448	Heavy metal-associated domain, HMA
	PEXP_103630	KGO36296	Urease accessory protein UreD
	PEXP_083550	KGO40992	Thiamine pyrophosphate enzyme, C-terminal TPP-binding
	PEXP_070880	KGO40584	Polyketide synthase, enoylreductase
	PEXP_027120	KGO42963	hypothetical protein
	PEXP_040610	KGO48229	Transcription factor, fungi
	PEXP_075350	KGO36016	Alpha-actinin
	PEXP_011040	KGO49055	Cytochrome P450, E-class, group I
	PEXP_005060	KGO36707	Transcription factor, fungi
	PEXP_070870	KGO40583	Aldehyde dehydrogenase, C-terminal
	PEXP_046450	KGO39171	Amidohydrolase 1
	PEXP_008360	KGO37036	4-Hydroxyphenylpyruvate dioxygenase
	PEXP_048620	KGO39686	Nickel/cobalt transporter, high-affinity
	PEXP_101160	KGO38158	Pectin lyase fold/virulence factor
	PEXP_022330	KGO42704	Transcription factor, fungi
	PEXP_058230	KGO38945	Alcohol dehydrogenase superfamily, zinc-type
	PEXP_053390	KGO42387	Polyketide synthase, enoylreductase
	PEXP_017020	KGO46060	Cytochrome P450
	PEXP_021500	KGO44639	Transcription factor, fungi
	PEXP_043440	KGO39263	Polyketide synthase, enoylreductase
	PEXP_102420	KGO36222	Protein of unknown function DUF3468
	PEXP_108970	KGO41891	Polyketide synthase, enoylreductase
	PEXP_095570	KGO43655	Transcription factor, fungi
	PEXP_088350	KGO41611	Transcription factor, fungi
	PEXP_056220	KGO38423	Cytochrome P450
	PEXP_099120	KGO46300	Hypothetical protein
	PEXP_028450	KGO43127	Transcription factor, fungi
	PEXP_110420	KGO38764	ATPase, P-type, K/Mg/Cd/Cu/Zn/Na/Ca/Na/H-transporter
	PEXP_050400	KGO42090	Zinc finger, C2H2
	PEXP_088420	KGO41618	ATPase, P-type, K/Mg/Cd/Cu/Zn/Na/Ca/Na/H-transporter
	PEXP_085230	KGO41160	Hypothetical protein
	PEXP_000560	KGO44365	Class II aldolase/adducin N-terminal
	PEXP_071150	KGO40611	Alcohol dehydrogenase superfamily, zinc-type
	PEXP_090470	KGO45017	Pyruvate carboxyltransferase
	PEXP_080790	KGO38063	hypothetical protein
	PEXP_026670	KGO42918	Transcription factor, fungi
	PEXP_084100	KGO41047	Transcription factor, fungi
	PEXP_102160	KGO38273	Casein kinase II, regulatory subunit
	PEXP_036970	KGO35909	Cytochrome P450, E-class, group I
	PEXP_032030	KGO39788	Hypothetical protein
	PEXP_089350	KGO41723	Transcription factor, fungi
	PEXP_005230	KGO36724	Transcription factor, fungi
	PEXP_057180	KGO44225	ATP adenylyltransferase, C-terminal
	PEXP_002560	KGO37249	Hypothetical protein
	PEXP_081210	KGO47322	ATP-grasp fold, subdomain 1
	PEXP_071070	KGO40603	Molybdenum cofactor synthesis C-terminal
	PEXP_095390	KGO43637	Transcription factor, fungi
	PEXP_054870	KGO44003	Transcription factor, fungi
	PEXP_042700	KGO39429	Hypothetical protein
	PEXP_036990	KGO35911	Ureohydrolase
	PEXP_041200	KGO48300	Transcription factor, fungi
	PEXP_050270	KGO42077	ATPase, P-type, K/Mg/Cd/Cu/Zn/Na/Ca/Na/H-transporter
	PEXP_048130	KGO39637	Transcription factor, fungi
	PEXP_010010	KGO48952	Transcription factor, fungi
	PEXP_085290	KGO41166	Transcription factor, fungi
	PEXP_079380	KGO37922	Zinc finger, C2H2
	PEXP_057190	KGO44226	Thiamine pyrophosphate enzyme, C-terminal TPP-binding
	PEXP_069040	KGO46734	Cytochrome P450
	PEXP_007560	KGO36956	Zinc finger, C2H2
	PEXP_081220	KGO47323	Transcription factor, fungi
	PEXP_087170	KGO40934	Transcription factor, fungi
	PEXP_105830	KGO41509	Transcription factor, fungi
	PEXP_043140	KGO39473	Cytochrome P450
	PEXP_067370	KGO46568	hypothetical protein
	PEXP_073070	KGO48577	Exonuclease, RNase T/DNA polymerase III
	PEXP_051020	KGO42152	Amidohydrolase 1
	PEXP_061400	KGO45512	Hypothetical protein
	PEXP_101010	KGO38143	Glycoside hydrolase, family 71
	PEXP_043150	KGO39474	Terpenoid synthase
	PEXP_015320	KGO47645	ATP-grasp fold, subdomain 1
Pectate lyase activity	PEXP_101160	KGO38158	Pectin lyase fold/virulence factor
	PEXP_080220	KGO38006	Pectate lyase, catalytic
Calcium ion transmembrane transporter activity	PEXP_046990	KGO39741	Sodium/calcium exchanger membrane region
	PEXP_088420	KGO41618	ATPase, P-type, K/Mg/Cd/Cu/Zn/Na/Ca/Na/H-transporter
	PEXP_095400	KGO43638	Sodium/calcium exchanger membrane region
	PEXP_050270	KGO42077	ATPase, P-type, K/Mg/Cd/Cu/Zn/Na/Ca/Na/H-transporter
Sequence-specific DNA binding RNA polymerase II transcription factor activity	PEXP_102680	KGO36423	Transcription factor, fungi
	PEXP_042010	KGO48399	Protein of unknown function DUF3468
	PEXP_095680	KGO43666	Protein of unknown function DUF3468
	PEXP_040610	KGO48229	Transcription factor, fungi
	PEXP_070870	KGO40583	Aldehyde dehydrogenase, C-terminal
	PEXP_021500	KGO44639	Transcription factor, fungi
	PEXP_102420	KGO36222	Protein of unknown function DUF3468
	PEXP_088350	KGO41611	Transcription factor, fungi
	PEXP_099120	KGO46300	Hypothetical protein
	PEXP_085230	KGO41160	Hypothetical protein
	PEXP_080790	KGO38063	Hypothetical protein
	PEXP_102160	KGO38273	Casein kinase II, regulatory subunit
	PEXP_032030	KGO39788	Hypothetical protein
	PEXP_005230	KGO36724	Transcription factor, fungi
	PEXP_057180	KGO44225	ATP adenylyltransferase, C-terminal
	PEXP_002560	KGO37249	Hypothetical protein
	PEXP_095390	KGO43637	Transcription factor, fungi
	PEXP_042700	KGO39429	Hypothetical protein
	PEXP_048130	KGO39637	Transcription factor, fungi
	PEXP_010010	KGO48952	Transcription factor, fungi
	PEXP_085290	KGO41166	Transcription factor, fungi
	PEXP_087170	KGO40934	Transcription factor, fungi
	PEXP_067370	KGO46568	Hypothetical protein
	PEXP_101010	KGO38143	Glycoside hydrolase, family 71
Hydrolase activity, hydrolyzing O-glycosyl compounds	PEXP_000940	KGO44403	Protein of unknown function DUF2985
	PEXP_044190	KGO39338	Aldolase-type TIM barrel
	PEXP_049330	KGO39518	Glycoside hydrolase, family 35
	PEXP_023030	KGO42774	Glycoside hydrolase, superfamily
	PEXP_101680	KGO38226	Concanavalin A-like lectin/glucanases superfamily
	PEXP_023340	KGO42463	Glycoside hydrolase, family 43
	PEXP_070780	KGO40574	Glycoside hydrolase, family 31
	PEXP_072880	KGO48558	Glycoside hydrolase, superfamily
	PEXP_048880	KGO39711	Concanavalin A-like lectin/glucanases superfamily
	PEXP_069330	KGO46763	Glycoside hydrolase, family 61
	PEXP_011880	KGO49139	Glycoside hydrolase, family 28
	PEXP_089320	KGO41720	Glycoside hydrolase, superfamily
	PEXP_076310	KGO37551	Glycoside hydrolase, family 32
	PEXP_026640	KGO42915	Glycoside hydrolase, family 16, CRH1, predicted
	PEXP_057570	KGO44264	Glycoside hydrolase family 3
	PEXP_053240	KGO42372	Glycoside hydrolase, family 61
	PEXP_042140	KGO48412	Concanavalin A-like lectin/glucanase, subgroup
	PEXP_107940	KGO41788	Alpha-amylase, C-terminal all beta
ATPase activity, coupled to transmembrane movement of substances	PEXP_023060	KGO42777	CDR ABC transporter
	PEXP_050880	KGO42138	ABC transporter, integral membrane type 1
	PEXP_110420	KGO38764	ATPase, P-type, K/Mg/Cd/Cu/Zn/Na/Ca/Na/H-transporter
	PEXP_040720	KGO48240	ABC transporter, integral membrane type 1
	PEXP_071990	KGO40700	ABC transporter, integral membrane type 1
	PEXP_088420	KGO41618	ATPase, P-type, K/Mg/Cd/Cu/Zn/Na/Ca/Na/H-transporter
	PEXP_104880	KGO41412	ABC transporter, integral membrane type 1
	PEXP_050270	KGO42077	ATPase, P-type, K/Mg/Cd/Cu/Zn/Na/Ca/Na/H-transporter
	PEXP_077310	KGO37715	CDR ABC transporter
	PEXP_086490	KGO40844	ABC transporter, integral membrane type 1
	PEXP_057530	KGO44260	ABC transporter, integral membrane type 1
4-Hydroxyphenylpyruvate dioxygenase activity	PEXP_084560	KGO41093	4-Hydroxyphenylpyruvate dioxygenase
	PEXP_008360	KGO37036	4-Hydroxyphenylpyruvate dioxygenase
Integral component of membrane	PEXP_046990	KGO39741	Sodium/calcium exchanger membrane region
	PEXP_024890	KGO42618	Major facilitator superfamily domain, general substrate transporter
	PEXP_041670	KGO48366	Major facilitator superfamily domain, general substrate transporter
	PEXP_059490	KGO45335	Sulfate anion transporter
	PEXP_043720	KGO39291	Alkali metal cation/H+ antiporter Nha1, C-terminal
	PEXP_052330	KGO42281	Major facilitator superfamily domain, general substrate transporter
	PEXP_037770	KGO36126	Major facilitator superfamily domain, general substrate transporter
	PEXP_102860	KGO36441	Mitochondrial carrier protein
	PEXP_023060	KGO42777	CDR ABC transporter
	PEXP_033600	KGO39977	Amino acid/polyamine transporter I
	PEXP_014370	KGO47550	Amino acid transporter, transmembrane
	PEXP_094860	KGO43584	Major facilitator superfamily domain, general substrate transporter
	PEXP_006100	KGO36811	Major facilitator superfamily domain, general substrate transporter
	PEXP_046430	KGO39169	Major facilitator superfamily domain, general substrate transporter
	PEXP_086200	KGO40808	Amino acid/polyamine transporter I
	PEXP_024440	KGO42573	Major facilitator superfamily domain, general substrate transporter
	PEXP_003610	KGO37353	Major facilitator superfamily domain, general substrate transporter
	PEXP_043410	KGO39260	Major facilitator superfamily domain, general substrate transporter
	PEXP_048620	KGO39686	Nickel/cobalt transporter, high-affinity
	PEXP_006360	KGO36837	Major facilitator superfamily domain, general substrate transporter
	PEXP_044620	KGO39381	Tetracycline resistance protein, TetA/multidrug resistance protein MdtG
	PEXP_072400	KGO48510	Major facilitator superfamily domain, general substrate transporter
	PEXP_028370	KGO43119	Amino acid transporter, transmembrane
	PEXP_009920	KGO48943	Peroxisomal biogenesis factor 11
	PEXP_033410	KGO39958	Major facilitator superfamily domain, general substrate transporter
	PEXP_050880	KGO42138	ABC transporter, integral membrane type 1
	PEXP_056980	KGO44205	Major facilitator superfamily domain, general substrate transporter
	PEXP_102420	KGO36222	Protein of unknown function DUF3468
	PEXP_074410	KGO48711	Major facilitator superfamily domain, general substrate transporter
	PEXP_068910	KGO46721	Major facilitator superfamily domain, general substrate transporter
	PEXP_060610	KGO45433	Major facilitator superfamily domain, general substrate transporter
	PEXP_009670	KGO49300	Cation/H+ exchanger
	PEXP_017290	KGO46087	Major facilitator superfamily domain, general substrate transporter
	PEXP_110420	KGO38764	ATPase, P-type, K/Mg/Cd/Cu/Zn/Na/Ca/Na/H-transporter
	PEXP_059360	KGO45322	Mitochondrial carrier protein
	PEXP_095790	KGO43677	Major facilitator superfamily domain, general substrate transporter
	PEXP_040720	KGO48240	ABC transporter, integral membrane type 1
	PEXP_071990	KGO40700	ABC transporter, integral membrane type 1
	PEXP_048590	KGO39683	Amino acid/polyamine transporter I
	PEXP_104220	KGO36561	Cation/H+ exchanger
	PEXP_000120	KGO44321	Major facilitator superfamily domain, general substrate transporter
	PEXP_019490	KGO45826	C4-dicarboxylate transporter/malic acid transport protein
	PEXP_088420	KGO41618	ATPase, P-type, K/Mg/Cd/Cu/Zn/Na/Ca/Na/H-transporter
	PEXP_076320	KGO37552	Major facilitator superfamily domain, general substrate transporter
	PEXP_001370	KGO37112	Major facilitator superfamily domain, general substrate transporter
	PEXP_001360	KGO37111	Major facilitator superfamily domain, general substrate transporter
	PEXP_104880	KGO41412	ABC transporter, integral membrane type 1
	PEXP_005240	KGO36725	Major facilitator superfamily domain, general substrate transporter
	PEXP_108540	KGO41848	Major facilitator superfamily domain, general substrate transporter
	PEXP_095400	KGO43638	Sodium/calcium exchanger membrane region
	PEXP_046610	KGO39187	Sodium/calcium exchanger membrane region
	PEXP_098820	KGO46270	Major facilitator superfamily domain, general substrate transporter
	PEXP_001950	KGO37170	Mitochondrial carrier protein
	PEXP_050270	KGO42077	ATPase, P-type, K/Mg/Cd/Cu/Zn/Na/Ca/Na/H-transporter
	PEXP_023180	KGO42789	Major facilitator superfamily domain, general substrate transporter
	PEXP_010400	KGO48991	Major facilitator superfamily domain, general substrate transporter
	PEXP_098540	KGO46242	Major facilitator superfamily domain, general substrate transporter
	PEXP_066250	KGO46934	Major facilitator superfamily domain, general substrate transporter
	PEXP_062420	KGO45644	General substrate transporter
	PEXP_026170	KGO42868	Major facilitator superfamily domain, general substrate transporter
	PEXP_077310	KGO37715	CDR ABC transporter
	PEXP_022750	KGO42746	Major facilitator superfamily domain, general substrate transporter
	PEXP_068690	KGO46699	Major facilitator superfamily domain, general substrate transporter
	PEXP_066860	KGO46517	Major facilitator superfamily domain, general substrate transporter
	PEXP_086490	KGO40844	ABC transporter, integral membrane type 1
	PEXP_067360	KGO46567	Major facilitator superfamily domain, general substrate transporter
	PEXP_057530	KGO44260	ABC transporter, integral membrane type 1
	PEXP_034320	KGO40048	Arsenical pump membrane protein, ArsB
	PEXP_074940	KGO36159	Major facilitator superfamily domain, general substrate transporter

A second cluster that was under-represented, both in colonized apple tissue and in vitro at pH 4 was cluster 8 (Fig. 2). This cluster mainly showed enrichment in carbon utilization and sugar-transport-related genes (Table 3, Additional file 7: Figure S8), which suggests that they contribute to a series of processes that induce carbon catabolism and thereby result in GLA accumulation [8]. This behavior may indicate that these processes are usually repressed during colonization unless there is a significant increase in ammonia accumulation.

**Table 3:** Genes involved in each process of cluster 8

GO Term	Gene id	Genbank transcript id	Description
Phosphate ion transport	PEXP_088430	KGO41619	Phosphate transporter
	PEXP_030290	KGO40453	Phosphate transporter
Carbon utilization	PEXP_105770	KGO41503	Ribose/galactose isomerase
	PEXP_105760	KGO41502	Aldolase-type TIM barrel
Transmembrane transport	PEXP_104850	KGO41409	Major facilitator superfamily domain, general substrate transporter
	PEXP_048020	KGO39626	Major facilitator superfamily domain, general substrate transporter
	PEXP_070650	KGO40561	Major facilitator superfamily domain, general substrate transporter
	PEXP_011530	KGO49104	Major facilitator superfamily domain, general substrate transporter
	PEXP_033090	KGO39926	Amino acid/polyamine transporter I
	PEXP_078910	KGO37875	Cation efflux protein
	PEXP_049390	KGO39524	Cation/H+ exchanger
	PEXP_030290	KGO40453	Phosphate transporter
	PEXP_034270	KGO40044	Major facilitator superfamily domain, general substrate transporter
Cation transport	PEXP_002160	KGO37191	ATPase, P-type, K/Mg/Cd/Cu/Zn/Na/Ca/Na/H-transporter
	PEXP_078910	KGO37875	Cation efflux protein
	PEXP_049390	KGO39524	Cation/H+ exchanger
	PEXP_004260	KGO37441	Sodium\x3aneurotransmitter symporter
Neurotransmitter transport	PEXP_004260	KGO37441	Sodium\x3aneurotransmitter symporter
Triose-phosphate isomerase activity	PEXP_105760	KGO41502	Aldolase-type TIM barrel
Ribose-5-phosphate isomerase activity	PEXP_105770	KGO41503	Ribose/galactose isomerase
Cysteine dioxygenase activity	PEXP_086730	KGO40868	Cysteine dioxygenase type I
asparaginase activity	PEXP_042000	KGO48398	L-asparaginase, type II
Integral component of membrane	PEXP_002160	KGO37191	ATPase, P-type, K/Mg/Cd/Cu/Zn/Na/Ca/Na/H-transporter
	PEXP_104850	KGO41409	Major facilitator superfamily domain, general substrate transporter
	PEXP_025010	KGO42630	Hypothetical protein
	PEXP_083360	KGO38643	FAD-binding 8
	PEXP_048020	KGO39626	Major facilitator superfamily domain, general substrate transporter
	PEXP_070650	KGO40561	Major facilitator superfamily domain, general substrate transporter
	PEXP_011530	KGO49104	Major facilitator superfamily domain, general substrate transporter
	PEXP_033090	KGO39926	Amino acid/polyamine transporter I
	PEXP_078910	KGO37875	Cation efflux protein
	PEXP_049390	KGO39524	Cation/H+ exchanger
	PEXP_004260	KGO37441	Sodium\x3aneurotransmitter symporter
	PEXP_034270	KGO40044	Major facilitator superfamily domain, general substrate transporter

### Clusters with similar expression levels in colonized apple tissue and in vitro at pH 7

#### High expression level of fungal genes in colonized apple tissue and in fungi grown in vitro at pH 7

**Fig. 3:**
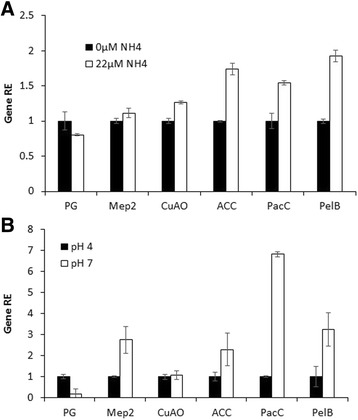
Effects of ammonia and pH on the relative expressions of genes involved in the pathogenicity process. Expressions were analyzed of: polygalacturonase (PG), Mep2, Copper amine oxidase (CuAO), ACC deaminase, PacC, and pectate lyase A (PelA). **a** Samples from the leading edge of infection in apples treated with exogenous NH_4_Cl at 0 and 22 μM, according to Barad et al. [8]; **b** Samples grown at pH 4 or pH 7, for 3 h in shaking secondary media (SM)

One of the most important clusters that showed high expression levels both in colonized apple tissue and in vitro at pH 7 was number 9 (Fig. 2), which showed significant enrichment of cellular amino-acid metabolism (Table 4, Additional file 8: Figure S9). Ammonia accumulation is induced under the limited-nutrient conditions present at the leading edge of the decay, in order to enable the pathogen to use a battery of pectolytic enzymes for tissue maceration [8]. Among the genes involved in cellular amino-acid metabolism process is glucose-methanol-choline oxidoreductase (GMC) (Table 4), whose families are clusters of FAD flavoprotein oxidoreductases-highly complex genes that contribute to several oxidation and reduction processes [29]. These enzymes include a variety of proteins, not all of which were present in cluster 9, such as choline dehydrogenase (CHD), which was present in clusters 1, 3, and 9, methanol oxidase (MOX), and cellobiose dehydrogenase, which was present in cluster 3; these are proteins that share a number of homologous regions that show sequence similarities. Since ammonia accumulation at the leading edge of the *Penicillium*-colonized tissue did not increase local pH [8], it is possible that the accumulated ammonia may activate gene expression, either directly or indirectly by generation of hydrogen peroxide and activation of RBOH in the killed cell [30]. To elucidate the role of ammonia, we analyzed the relative expression levels of several genes-MepB, CuAC and ACC-from cluster 9 in *Penicillium*-colonized fruits exposed to 22 μM of NH_4_^+^, and found induction of their relative expression levels (Fig. 3), which indicates the capability of ammonia to activate this process.

**Table 4:** Genes involved in each process of cluster 9

GO Term	Gene id	Genbank transcript id	Description
Ammonium transmembrane transport	PEXP_025350	KGO42664	Ammonium transporter
Cellular amino acid metabolic process	PEXP_082650	KGO38572	Pyridoxal phosphate-dependent transferase, major region, subdomain 2
	PEXP_109810	KGO38703	Glucose-methanol-choline oxidoreductase
	PEXP_004920	KGO36657	Glutamate synthase, central-N
	PEXP_053110	KGO42359	Catalase-peroxidase heme
	PEXP_056170	KGO38539	1-aminocyclopropane-1-carboxylate deaminase
Amine metabolic process	PEXP_053110	KGO42359	Catalase-peroxidase heme
	PEXP_056170	KGO38539	1-aminocyclopropane-1-carboxylate deaminase
	PEXP_000620	KGO44371	Copper amine oxidase, N2-terminal
Hydrogen peroxide catabolic process	PEXP_053110	KGO42359	Catalase-peroxidase heme

**Fig. 4:**
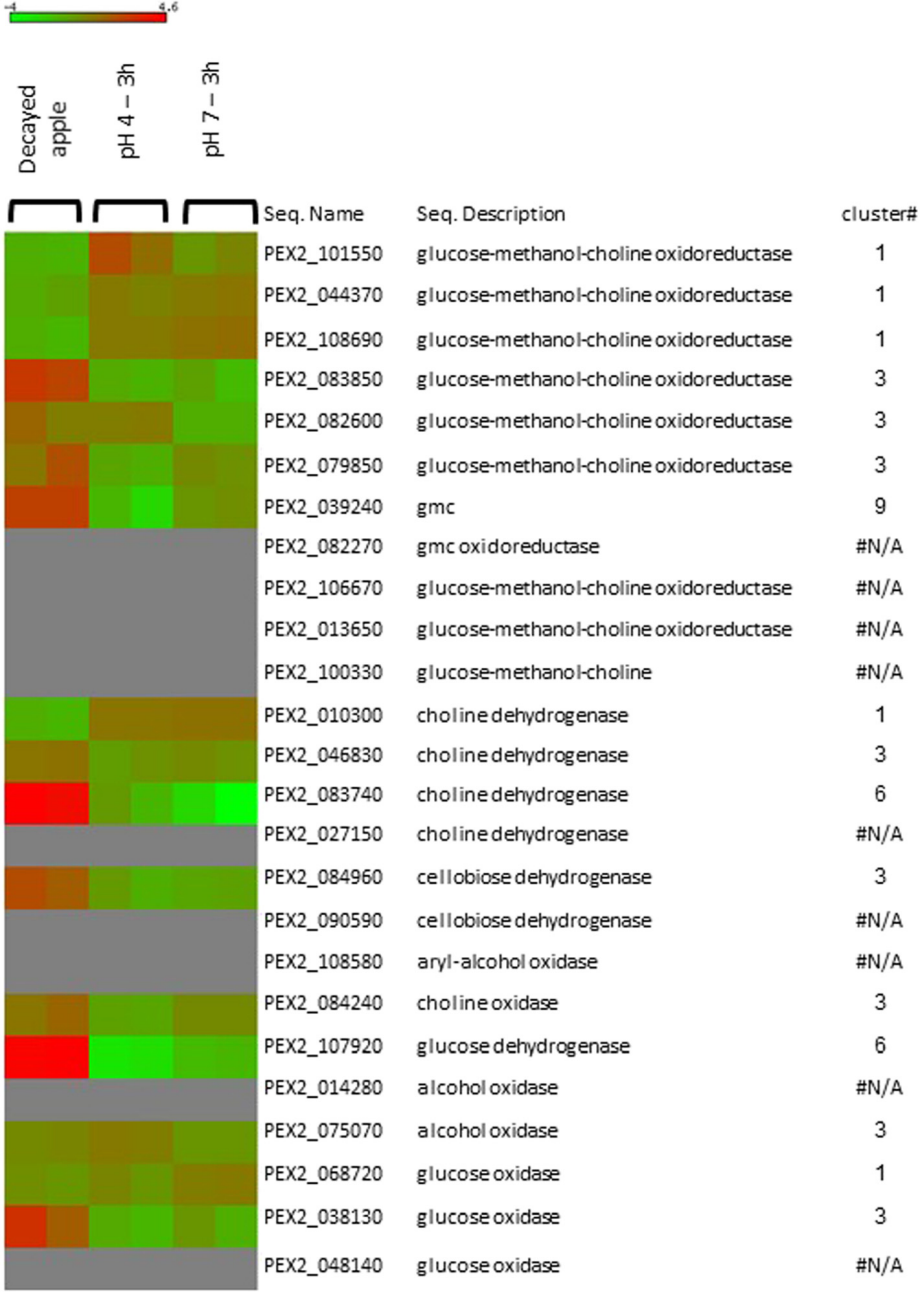
Heat map of the expression pattern of GMC oxidoreductase gene family. The expression patterns of genes belonging to the GMC oxidoreductase gene family are shown as a heat map obtained with matrix2png software [72]. Gene identity, gene description, and its location in the differentially expressed gene clusters are indicated

Another highly expressed family of genes in cluster 9 was the glucose-methanol-choline oxidoreductase family. Heat-map analysis of this family showed that genes with the same activity were differentially expressed between different clusters (Fig. 4). For example, 11 GMC oxidoreductase genes were detected in *P. expansum*: three in cluster 1, three in cluster 3, and one in cluster 9, whereas the remaining four were not differentially expressed. The GMC oxidoreductase family also exhibited close similarity to the glucose oxidase family. We were able to detect three glucose oxidase transcripts, as described by Ballester et al. [12], which showed differing expression patterns: gox1 of *P. expansum* was not differentially expressed under our conditions, *gox2* was found in cluster 3, and *gox3* was detected in cluster 1 (Fig. 4). Analysis of only the expression pattern at pH 4 compared with that at pH 7 showed that both *gox2* and glucose oxidase 3 (*gox3)* were upregulated at pH 7 but not at pH 4 [7]. This differential expression pattern of the *gox* family suggests that genes were being selectively activated on the basis of their optimal conditions with respect to pH, in vitro, or in vivo, to enable the fungus to cope with varied conditions and to make optimal use of the inventory of available enzymes [31].

Another process that was enriched in this cluster was amine metabolism (Additional file 8: Figure S9), which involves the gene copper amine oxidase (CuAO) (Table 4). In plants, wounding of tissue usually results in an increase in the steady-state levels of copper amine oxidase expression and H_2_O_2_ accumulation [32]. Activation of CuAO during *Penicillium* attack also may lead to enhanced accumulation of H_2_O_2_ at the wound site, thereby contributing to extension of necrotic lesions and extensive plant-cell damage [32]. Also, copper amine oxidase may catalyze oxidation of the aliphatic diamines putrescine and cadaverine at their primary amino groups [33]. The products of putrescine oxidation by CuAO are H_2_O_2_, NH_3_, and Δ1-pyrroline; and Δ1-pyrroline is further catabolized to γ-aminobutyric acid, which subsequently is transaminated and oxidized to succinic acid. Thus, in *Penicillium* copper amine oxidase may contribute to the balance of reactive oxygen species (ROS) produced in the cell wall extracellular matrix [34]. This specific regulatory mechanism and the molecular signals inducing modulation of copper amine oxidase in *Penicillium* during decay highlight the relevance of these enzymes as an H_2_O_2_-delivering system in colonized tissue. The overrepresentation of glucose-methanol-choline oxidoreductase- and copper amine oxidase-activated genes in cluster 9 may indicate their contribution to the ROS and oxidoreductase process at the leading edge of the colony; also, in the same cluster there was activation of the H_2_O_2_ catabolic process (Additional file 8: Figure S9) through catalase peroxidase, which suggests a hitherto unknown mechanism of *Penicillium* survival under oxidative stress. Interestingly, similar up-regulation of catalase peroxidase under pathogenic conditions was reported to account for the survival of *P. marneffei*, an intracellular pathogen that causes common opportunistic infections in humans, and of *P. simplicissimum*, a plant pathogen in which strong expression of the catalase peroxidase transcripts may contribute to survival of this fungus in host cells [35]. Considered together, the relative expressions of the oxidoreductase genes and copper amine oxidase under fruit pH levels ranging from 3.7 to 4.2, again may indicate the importance of local ammonification, as reported by Barad et al. [8], as a mechanism to induce activation of genes usually overexpressed at pH 7 (Fig. 3).

The overrepresentation in cluster 9, of several nitrogen-metabolism-regulating genes, such as glutamate synthase and MepB (Table 4) also is important for nitrogen metabolism in fungi. The ammonia transporter encoded by *mepB* can lead to an internal/external modulation of ammonia in the hypha, activation of *pacC*, and high-pH induced genes [36]. Accumulation of glutamate, followed by its transformation to glutamine by glutamate synthase activity may be the basis for accumulation of several amino acids, and both stages are activated by ammonia accumulation (Fig. 3). Overall, the nitrogen mobilization promoted by infection could be considered as part of a "slash-and-burn" strategy that deprives the pathogen of nutrients and modulates alkaline-expressed genes. Such nutritional and metabolic changes might occur as a differential-attack mechanism promoting pathogen development [37].

One interesting gene also activated in cluster 9 in cellular amino acid metabolism (Additional file 8: Figure S9) is the gene encoding for aminocyclopropane-1-carboxylate deaminase (ACC) (Table 4). The ACC functions as a deaminase, degrading aminocyclopropane-1-carboxylate deaminase to 2-oxobutyrate and ammonia, which is a precursor of the plant hormone ethylene. A similar reaction process catalyzing ACC synthase was found in *Penicillium digitatum* attacking citrus fruits [38, 39], which suggests that colonization may be associated with ethylene production and induced tissue senescence. Taken together these results indicate that ROS production by the pathogen may be accompanied by host tissue induced senescence.

#### Low expression level of fungal genes in colonized apple tissue and in fungus grown in vitro at pH 7

Group III also includes cluster 4, in which colonized apple tissue showed under-representation of transcripts similar to that in vitro at pH 7 (Fig. 2). This cluster shows enrichment of genes involved in the oxido-reduction process, such as cysteine dioxygenase (Table 5, Additional file 9: Figure S4). Among representatives of this group are the cytochrome P-450 (CytP) monooxygenases, which are enzymes that: catalyze conversion of hydrophobic intermediates of primary and secondary metabolic pathways; detoxify natural and environmental pollutants that are accumulated during colonization as phytochemical molecules belonging to the polyphenols; and enable fungal growth under varied colonizing conditions [40]. They do this by inserting one oxygen atom into the aliphatic position of an organic substrate, while the other oxygen atom is reduced to water.

**Table 5:** Genes involved in each process of cluster 4

GO Term	Gene id	genbank transcript id	Description
Oxidation-reduction process	PEXP_035940	KGO40210	Cytochrome P450, E-class, CYP52
	PEXP_094610	KGO43560	Multicopper oxidase, type 1
	PEXP_069560	KGO47265	Cytochrome P450
	PEXP_047900	KGO39614	Cytochrome P450
	PEXP_074010	KGO48671	Cytochrome P450
	PEXP_065530	KGO46863	Short-chain dehydrogenase/reductase SDR
	PEXP_073620	KGO48632	Polyketide synthase, enoylreductase
	PEXP_079160	KGO37900	FAD-linked oxidase, N-terminal
	PEXP_057820	KGO44289	Cysteine dioxygenase type I
	PEXP_043230	KGO39482	Isocitrate and isopropylmalate dehydrogenases family
	PEXP_101170	KGO38159	Oxoglutarate/iron-dependent dioxygenase
	PEXP_060240	KGO45235	Short-chain dehydrogenase/reductase SDR
	PEXP_034900	KGO40106	Aldolase-type TIM barrel
	PEXP_106700	KGO41290	Isocitrate and isopropylmalate dehydrogenases family
	PEXP_026690	KGO42920	Short-chain dehydrogenase/reductase SDR
	PEXP_069740	KGO47756	Redoxin
	PEXP_103230	KGO36363	Cytochrome P450
	PEXP_091400	KGO45110	hypothetical protein
	PEXP_027810	KGO43163	Polyketide synthase, enoylreductase
	PEXP_023550	KGO42484	Indoleamine 2,3-dioxygenase
	PEXP_077820	KGO37766	Polyketide synthase, enoylreductase
	PEXP_043790	KGO39298	Dihydrolipoamide succinyltransferase
	PEXP_082800	KGO38587	Cytochrome P450
	PEXP_043420	KGO39261	Polyketide synthase, enoylreductase
	PEXP_030200	KGO40444	Cytochrome P450
	PEXP_061260	KGO45498	Short-chain dehydrogenase/reductase SDR
	PEXP_078860	KGO37870	Dimeric alpha-beta barrel
	PEXP_042100	KGO48408	NADPH-cytochrome p450 reductase, FAD-binding, alpha-helical domain-3
	PEXP_037510	KGO35963	Oxoglutarate/iron-dependent dioxygenase
	PEXP_010380	KGO48989	Aldo/keto reductase
	PEXP_000410	KGO44350	Acyl transferase/acyl hydrolase/lysophospholipase
	PEXP_076770	KGO37511	3-oxo-5-alpha-steroid 4-dehydrogenase, C-terminal
	PEXP_066880	KGO46519	FAD dependent oxidoreductase
	PEXP_008580	KGO37058	hypothetical protein
	PEXP_001620	KGO37137	Ubiquinone biosynthesis protein Coq7
	PEXP_030860	KGO40271	NADPH-dependent FMN reductase
	PEXP_107740	KGO41768	Polyketide synthase, enoylreductase
	PEXP_049110	KGO39496	Monooxygenase, FAD-binding
	PEXP_017320	KGO46090	Polyketide synthase, enoylreductase
Taurine metabolic process	PEXP_057820	KGO44289	Cysteine dioxygenase type I
	PEXP_050220	KGO42072	Acetate/Proprionate kinase

### Carbohydrate active enzymes (CAZy) cluster distribution

The diverse complex carbohydrates that contribute to *Penicillium* maceration capabilities are controlled by a panel of enzymes involved in their assembly (glycosyltransferases) and breakdown (glycoside hydrolases, polysaccharide lyases, carbohydrate esterases), which collectively are designated as Carbohydrate-Active enZymes (CAZymes) [41]. In plant pathogens CAZymes promote synthesis, degradation, and modification of carbohydrates that play important roles in the breakdown of plant cell walls and in host/pathogen interactions [42]. *Penicillium* uses these enzymes to macerate the colonized tissue by degradation of complex carbohydrates of the hosts to simple monomers that can be utilized as nutrients [43, 44].

**Fig. 5:**
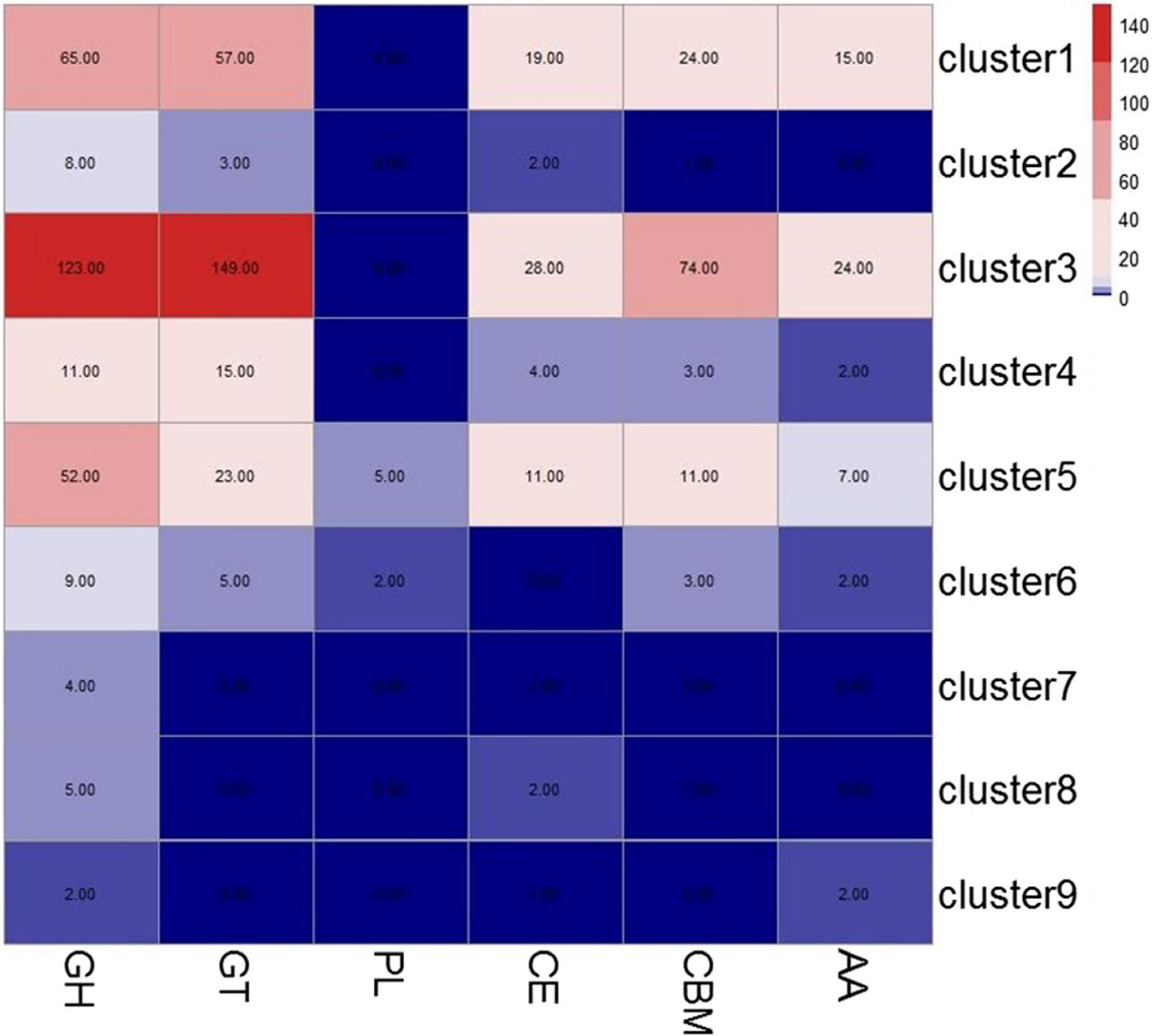
Comparison of the CAZyme repertories identified in each cluster of co-expressed genes. Enzyme families are represented by their class (GH-glycoside hydrolases; GT-glycosyltransferases; PL-polysaccharide lyases; CE-carbohydrate esterases; and CBM-chitin-binding modules) and family number according to the Carbohydrate-Active Enzyme Database. Abundance of the various enzymes within a family is represented on a color scale from 0 (dark blue) to 150 occurrences (dark red) per cluster

The CAZymes analysis toolkit (CAT) [45] was used to identify 771 putative CAZymes in *P. expansum* within the various clusters (Fig. 5). CAZymes formed 8.00 % of all the transcripts in cluster 1, with 15, 24, 19, 57, and 65 of CAZymes families including auxiliary activity (AA), carbohydrate-binding modules (CBM), carbohydrate esterases (CE), glycosyltransferases (GT), and glycoside hydrolases (GH), respectively. In cluster 2 they formed 5.57 % of all the transcripts with 2, 3, and 8 of CAZymes families including carbohydrate esterases, glycosyltransferases (GT), and glycoside hydrolases (GH), respectively. In cluster 3 they formed 17.92 % of all the transcripts, with 24, 74, 28, 149, and 123 of CAZymes families including auxiliary activity (AA), carbohydrate-binding modules (CBM), carbohydrate esterases (CE), glycosyltransferases (GT), and glycoside hydrolases (GH), respectively. In cluster 4 they formed 14.46 % of all transcripts, with 2, 3, 4, 15, and 11 of CAZymes families including including auxiliary activity (AA), carbohydrate-binding modules (CBM), carbohydrate esterases (CE), glycosyltransferases (GT), and glycoside hydrolases (GH), respectively. In cluster 5 they formed 22.61 % of all transcripts, with 7, 11, 11, 5, 23, and 52 of CAZymes families including auxiliary activity (AA), carbohydrate-binding modules (CBM), carbohydrate esterases (CE), polysaccharide lyases (PL), glycosyltransferases (GT), and glycoside hydrolases (GH), respectively. In cluster 6 they formed 29.16 % of all transcripts, with 2, 3, 2, 5, and 9 of CAZymes families including auxiliary activity (AA), carbohydrate-binding modules (CBM), PL, glycosyltransferases (GT), and glycoside hydrolases (GH), respectively. In cluster 7 they formed 22.61 % of all transcripts, with 4 of CAZymes families including glycosyl hydrolases. In cluster 8 they formed 12.06 % of all transcripts, with 2 and 5 of CAZymes families including carbohydrate esterases (CE) and glycoside hydrolases (GH), respectively. In cluster 9 they formed 16.67 % of all transcripts, with 2 of CAZymes families including each of auxiliary activity (AA) and glycoside hydrolases (GH) (Fig. 5).

Carbohydrate esterases, glycoside hydrolases, and polysaccharide lyases are associated with the ability to utilize the diversity of carbohydrates present in the environment and within host fruits. Glycosyltransferases are mainly involved in the basal activities of fungal cells, e.g., fungal-cell-wall synthesis, glycogen cycle, and trehalose cycle [41, 44, 46]. The wide occurrence of CAZymes in the various *Penicillium* clusters, taken together with their importance in degradation of the plant cell wall, indicates their basic contribution to colonization of the host fruit.

### Differentially expressed genes in Penicillium expansum- and Colletotrichum gloeosporioides-infected apple compared with healthy tissue

In order to analyze the effect of *Penicillium expansum* infection during acidification on the host (apple tissue) response, we used RNAseq to analyze the differentially expressed genes induced by *P. expansum* infection. Overall, in the *P. expansum*-infected apple tissue we found 4,292 differentially expressed genes with FDR threshold < 0.001 and with expression levels increasing or decreasing by a factor greater or less than 8, respectively, i.e., greater or less than +3 or −3, respectively, on a logarithmic (base 2) scale. We found 2,427 up-regulated and 1,865 down-regulated genes in colonized apple tissue. Analysis of the enriched pathways with MatGeneMap [47] revealed 21 significantly up-regulated pathways (Table 6) and 21 down-regulated ones (Table 7) with FDR < 0.05. Among the significantly up-regulated processes induced by *Penicillium* in apple tissue were: the jasmonic acid (JA), the mevalonate, and the flavonoid biosynthesis pathways, and the geranyl geranyldiphosphate biosynthesis I super pathway. Among the down-regulated pathways were the glycogen biosynthesis I and the starch biosynthesis pathways. Jasmonic acid is a lipid-derived signaling compound involved in regulation of diverse processes in plants such as fruit ripening, root growth, tendril coiling, senescence, and resistance to pathogens [48]; JA and related compounds are synthesized in plants via the octadecanoid pathway [49]. Biosynthesis of jasmonates starts with oxygenation of linolenic acid, which is thought to be released from membrane lipids through the action of a lipase, followed by several oxidation processes. This activation may indicate that there is a host response prior to fungal maceration induced by *Penicillium*, and this supposition is supported by findings that fruits pretreated with JA and related compounds showed enhanced resistance to pathogens [50].

**Table 6:** Apple up-regulated pathways by infection with *P. expansum*

Pathway name	*p* value (adjusted)
Jasmonic acid biosynthesis	5.73E-11
Mevalonate pathway	9.12E-08
Flavonoid biosynthesis	7.62E-05
Superpathway of geranylgeranyldiphosphate biosynthesis I (via mevalonate)	7.62E-05
Glutathione-mediated detoxification	0.00171
Trans,trans-farnesyl diphosphate biosynthesis	0.0032
Glutamate dependent acid resistance	0.0032
Chorismate biosynthesis	0.00325
Amygdalin and prunasin degradation	0.00988
Salicylate biosynthesis	0.01194
DIMBOA-glucoside degradation	0.01415
β-Alanine biosynthesis II	0.01456
Acetate formation from acetyl-CoA II	0.01456
Pyruvate fermentation to acetate III	0.01456
Glutamate degradation III (via 4-aminobutyrate)	0.01693
Superpathway of phenylalanine and tyrosine biosynthesis	0.01693
Phospholipid desaturation	0.03878
Glycolipid desaturation	0.03878
13-LOX and 13-HPL pathway	0.04864
Divinyl ether biosynthesis II (13-LOX)	0.04864
Superpathway of phenylalanine, tyrosine, and tryptophan biosynthesis	0.04864

The analysis was performed with MetGenMap

**Table 7:** Apple down-regulated pathways by infection with *P. expansum*

Pathway name	*p* value (adjusted)
Glycogen biosynthesis I (from ADP-D-Glucose)	9.064E-08
Starch biosynthesis	6.194E-07
C4 Photosynthetic carbon assimilation cycle	0.0002403
UDP-galactose biosynthesis (salvage pathway from galactose using UDP-glucose)	0.0008945
Lipoate biosynthesis and incorporation I	0.0019429
Methylerythritol phosphate pathway	0.0031538
Acyl carrier protein metabolism	0.0034133
Trans-lycopene biosynthesis	0.0059809
Fatty acid biosynthesis initiation I	0.0070497
Pyridoxal 5'-phosphate biosynthesis	0.0087438
Superpathway of pyridoxal 5'-phosphate biosynthesis and salvage	0.0132283
Xylitol degradation	0.0161255
Starch degradation	0.0173149
Heme biosynthesis I	0.0178181
Heme biosynthesis from uroporphyrinogen I	0.0195451
Colanic acid building blocks biosynthesis	0.0199615
Xylose degradation I	0.0284483
Sucrose biosynthesis	0.0303525
5-Aminoimidazole ribonucleotide biosynthesis II	0.0332884
Biotin-carboxyl carrier protein	0.0357646
Methanol oxidation to formaldehyde	0.0437164

The analysis was performed with MetGenMap

The second process that was activated was the mevalonate pathway, which produces isoprenoids that are vital for diverse cellular functions; these isoprenoids include sterols, carotenoids, chlorophyll, plant hormones, and defense isoprenoids. The penetration of *Penicillium* probably induces defense isoprenoids such as were found in damaged plant leaves [51]. These comprise a wide variety of defense-related genes, including those that activate biosynthesis of JA and ethylene, as a possible response to pathogen penetrations, as was reported for *Botrytis cinerea* [52]. qRT-PCR analysis of the ethylene-responsive transcription factor 2-like showed a significant induction of this gene in the apple tissue as a result of the *Penicillium* infection (Table 8).

**Table 8:** Relative expression (Pe/Cg) of selected genes in apple tissue infected with *P. expansum* or *C. gloeosporioides*

Apple down-regulated genes	Apple (Pe)/apple (Cg)	Apple up-regulated genes	Apple (Pe)/apple (Cg)
Programmed cell death protein 4-like	0.025/0.112 = 0.223	Histone deacetylase hdt3-like	0.70/1.21 = 0.583
Auxin-repressed kda isoform x1	0.002/0.009 = 0.222	Phenylalanine ammonia-lyase 1	9.41/3.38 = 2.781
ap2-like ethylene-responsive transcription factor at2g41710 isoform x2	0.015/0.063 = 0.238	Chalcone synthase	16.33/24.23 = 0.670
udp-glucose:glycoprotein glucosyltransferase	0.104/0.291 = 0.357	Peroxidase 47	98.83/10.55 = 9.368
Senescence-associated carboxylesterase 101-like	0.079/0.192 = 0.411	Respiratory burst oxidase homolog protein d-like	185.14/24.15 = 7.664
1-Aminocyclopropane-1-carboxylate oxidase 1	0.034/0.064 = 0.544	Lipoxygenase	490.05/205.74 = 2.381
Anthocyanidin 3-o-glucosyltransferase 5-like	0.007/0.041 = 0.169	Indole-3-acetic acid-induced protein arg2	9.97/6.49 = 1.535
Expansin 1	0.002/0.040 = 0.067	Zinc finger an1 domain-containing stress-associated protein 12-like	17.66/17.78 = 0.993
ap2-like ethylene-responsive transcription factor at2g41710 isoform x2	0.012/0.045 = 0.282	NADPH--cytochrome p450 reductase isoform x2	8.18/4.04 = 2.022
Lysine-specific histone demethylase 1 homolog 1-like	0.075/0.192 = 0.394	Ethylene-responsive transcription factor 2-like	33.81/33.83 = 0.999
		Serine threonine-protein kinase-like protein ccr4	11.12/1.83 = 6.049

The relative expression of the apple genes was compared with healthy tissue

The third up-regulated process was related to biosynthesis of flavonoids, which are linked to fungal potential cytotoxicity and capacity to interact with enzymes through protein complexation [53]. Some flavonoids provide stress protection, for example, by acting as scavengers of free radicals such as reactive oxygen species (ROS), as reported by Falcone Ferreyra et al. [53]. During *P. expansum* pathogenicity, the fungus produces gluconic acid, with H_2_O_2_ as a by-product. The host reacts to ROS by activating the flavonoids biosynthesis pathway and thereby initiating a resistance response to fungal penetration. Production of secondary metabolites such as anthocyanins, isoflavonoids, and flavonol glycosides may contribute to this resistance. Plants such as Arabidopsis respond to the combination of biotic, i.e., bacterial, and abiotic, e.g., UV-B radiation, stresses through synthesis of defense-related compounds such as phytoalexins and lignin, which serve as structural barriers that restrict the spread of pathogens. These responses modify the expression of genes involved in the production of protective metabolites such as flavonols [53]. This behavior suggests that there is a significant apple response to *Penicillium* penetration under these susceptible conditions. This activation matches the fourth activated pathway of *P. expansum* colonization, in which the geranyl-geranyl diphosphate-mediated processes are activated, probably in biosynthesis of essential compounds such as chlorophylls, carotenoids, tocopherols, plastoquinones, and gibberellins, but mainly in production of a variety of secondary metabolites. All of which indicates the significant response of apple fruits in coping with the colonization process.

Two significantly down-regulated processes that modulate carbohydrate metabolism were found: glycogen biosynthesis I from ADP-D-glucose, and starch biosynthesis. Glycogen and starch are both multibranched polysaccharides of glucose that serve as a means of energy storage for fungi; their downregulation may indicate their importance as energy stores that rapidly can be mobilized from the cytosol/cytoplasm and that perform an important function during glucose consumption by *Penicillium* during the acidification process [7]. Down-regulation of these processes probably results from catabolism of the substrates during attack by *P. expansum* and strongly supports previous findings of Prusky et al. [3], Hadas et al. [4], and Barad et al. [7] that indicate acidification of the tissue as a factor in pathogenicity of *P. expansum*.

In order to analyze the responses of apple fruits to fungal acidification or alkalization, the differential expressions of specific genes induced during colonization by *Penicillium* were compared to those expressed during colonization of the same host by *Colletotrichum*. For that purpose, the relative expressions of 21 specific key genes, of which 10 were up-regulated and 11 down-regulated by *Penicillium* (Table 8), were compared in apple RNA extracted from the leading edges of tissue colonized by the two respective pathogens. Genes that were up-regulated in the fruit showed the same response pattern when fruits were colonized by either *Penicillium* or *Colletotrichum*, but the relative expression obtained in *Penicillium*-inoculated apples was always significantly higher than that in *Colletotrichum*-inoculated apples: the ratio of *P. expansum*: *C. gloeosporioides* relative expression responses ranged from 0.58 to 9.36. This indicates a stronger host response to *Penicillium* colonization than to *Colletotrichum* colonization. Analysis of the down-regulated genes also showed similar patterns of host responses to the two respective pathogens, but lower ratios of *P. expansum*: *C. gloeosporioides* relative expressions, ranging from 0.067 to 0.54, which indicates that, although the response patterns were similar, the alkalizing pathogen induced lower expression levels. These results indicate that host responses to *Penicillium* and to *Colletotrichum* attack did not show opposite senses of gene modulation, as might be expected from their contrasting pH modulation directions, but only reduced expression levels in the *Colletotrichum*-inoculated apples.

## Conclusions

Overall, gene-expression profiling of pH-dependent genes of *P. expansum* during colonization of apple fruits revealed three major effects: (1) a pattern of high gene expression during colonization that had no connection with pH response; (2) gene expression patterns during colonization similar to those obtained under in vitro growth conditions at pH 4; and (3) gene expression patterns during colonization similar to those obtained under in vitro growth conditions at pH 7. These three main trends indicate the existence of pH-regulated genes, expressed at pH 4 and pH 7 that may contribute to *P. expansum* colonization. One of the key processes overrepresented in cluster 7 at pH 4 was high expression of glutamate decarboxylase (GAD). One of the key products of this enzyme-GABA-largely originates from decarboxylation of L-glutamate [54], and it had been found to function in communication between tomato (*Lycopersicon esculentum* var. *commune* Bailey) plants and the fungus *Cladosporium fulvum* [55]. Hyphae of *C. fulvum* are restricted to the apoplast, therefore the fungus is dependent on the contents of the apoplastic nutrients. During infection, the GABA concentration in the apoplast increased from about 0.8 mM to 2–3 mM; this can be attributed to stimulation of glutamate dehydrogenase activity by decreased pH and increased cytosolic calcium, which are associated with pathogen attack [56]. In the present study, similar conditions were present in the *Penicillium*/apple interactions, where acidification by GLA accumulation may enhance the consumption of this amino acid.

Accumulation of ammonia, induced under the limited-nutrient conditions present at the edge of the decaying tissue, may contribute to gene expression, either by alkalization or by direct induction of ammonia during gene activation [8]. Modulation of ammonia levels by MepB transporters and/or the amine metabolic process (both overrepresented at pH 7 and decayed tissue in cluster 9) may contribute to the alkalizing effects and pH increase. Under these pH conditions also an over-representation of glucose-methanol-choline (GMC) oxidoreductase was observed. GMC shows similar activity to the GOX of *P. expansum* that contributed to oxidation and reduction processes [29] by providing the H_2_O_2_ required by ligninolytic peroxidases in *Pleurotus* species [57]. This combination of GMC genes [58], described here for the first time in *P. expansum* under alkaline conditions, indicates activity of new undescribed mechanisms that contribute to cell-wall degradation by generation of H_2_O_2_ during fungal colonization at pH 7.

In addition, the copper amino oxidase (CuAO) contributes to the enhanced accumulation of H_2_O_2_ at the wound site which, in turn, contributes to the extended necrotic lesions and extensive plant cell damage [32]. The CuAO catalyzes oxidation of aliphatic diamines of the primary amino groups that contribute to H_2_O_2_ and NH_3_ accumulation, which emphasizes the relevance of the H_2_O_2_-delivering system in colonized tissue [33]. This over-representation of GMC and CuAO indicates the contribution to ROS production and oxidoreductase processes at the leading edge of the developing colony, and attributes the presence of fungal catalase peroxidases the need to protect colonizing hyphae.

These oxidative processes were concurrent with activation of Cyt P-450 monoxygenase-in group 3, cluster 4-which catalyzes the conversion of hydrophobic intermediates of primary and secondary metabolic pathways, thereby detoxifying natural and environmental pollutants and allowing fungi to grow under difficult oxidative conditions [40], which accounts for the activation of genes coding for proteases, cell-wall-related enzymes, redox homoeostasis, and detoxification processes that are expressed during the infection process.

Exploiting these strong oxidative conditions during its necrotrophic development, the fungus further advances the colonization process by activation of aminocyclopropane-1-carboxylate deaminase (ACC) (cluster 9), a precursor of the plant hormone ethylene. A similar reaction process catalyzing ACC synthase was found when *P. digitatum* attacked citrus fruits [38, 39], which indicates that the colonization process involves induction of senescence of the colonized tissue, in conjunction with the strong oxidative process.

Out of the 771 putative CAZymes identified in *P. expansum,* eight were found to be expressed in clusters 7 and 9, which were the most important clusters modulating pathogenicity (Fig. 5). Given the importance of CAZymes during cell-wall degradation, it is clear that they contribute strongly to the apple maceration process. The present transcriptome analysis showed induced ammonification, and strong oxidative and senescence processes, accompanied by strong activation of pectolytic enzymes, all of which indicate the pH dependence of the tools used by the pathogen to colonize the environment. All these induced metabolic changes indicate the significant role of pH modulation in the pathogenicity of *P. expansum* in fruits.

Analysis of the fruit transcriptome suggested that even if apple fruits are susceptible to *Penicillium*, the fungus activates significant gene processes related to fruit resistance. This may indicate that the necrotrophic maceration of the tissue occurs, not as a senescence response, but as an active fungal process that supports its pathogenicity. Also interesting is the stronger response of *Penicillium* than of *Colletotrichum*, which indicates that the pH adjustment effected by the fungus did not affect the pattern but the level of host response. At the same time, these findings indicate that the fungal matching to the host is the main factor activating the maceration process.

## Methods

### Fungal strains and culture conditions

The WT *P. expansum* isolate Pe-21 was obtained from decayed apples (*Malus domestica* cv. Golden Delicious) purchased from a local market in Israel [4]. Cultures were grown at 27 °C in the dark, and maintained on PDA plates (Difco, MD, USA) unless otherwise indicated. Conidia were harvested with 10 mL of sterile distilled water supplemented with 0.01 % (v/v) Tween 80 (Sigma-Aldrich, Copenhagen, Denmark). Cells were visualized with a model BX60F-3 microscope and a model SZ-60 stereoscope (both from Olympus America Inc., Melville, NY, USA) and counted with a hemocytometer (Brand, GMBH, Werheim, Germany).

### Assay for colonization and disease development

'Golden Delicious' apples were inoculated by placing 5 μL of a conidial suspension containing 10^6^ spores mL^−1^ on each of six 2-mm-deep, 2-mm-diameter wounds spaced evenly in a circle around the upper part of the stem end of the fruits. Following inoculation, the fruits were incubated for 5 days at 25 °C in covered plastic containers containing wet paper towels. Samples of healthy tissue and of the leading edge of each wound were collected and frozen with liquid nitrogen for subsequent RNA analysis.

### In vitro procedure of P. expansum

Fungal spores were inoculated at 10^6^ spores mL^−1^ into 40 mL of a primary medium, i.e., glucose minimal medium, in 125-mL flasks containing (per liter) 10 g sucrose, 5 g yeast extract (Difco Laboratories, MD, USA), 50 mL nitrate salts, and 1 mL trace elements, at pH 4.5. The cultures were incubated at 25 °C with shaking at 150 rpm for 48 h. Cultures were harvested by vacuum filtration through a sterile Büchner funnel fitted with a Whatman number 1 filter paper, and the remaining mycelia were washed twice with 50 mL of sterile distilled water. The washed mycelia were resuspended in 50 mL of 0.2 M phthalate-buffered or phosphate-buffered liquid secondary medium (SM) at pH 4 or pH 7, respectively. This SM contained (per liter) 60 g sucrose, 7 g NaNO_3_, 3 g tryptone (Difco Laboratories, MD, USA), 1 g KH_2_PO_4_, 0.5 g MgSO_4_ · 7H_2_O, and 0.5 g KCl. The cultures were incubated at 25 °C on a rotating shaker at 150 rpm for 0.5, 1, 3, 10, or 24 h. A sample of mycelia from the cultures was collected into a 1.5-mL Eppendorf at each time point, and the mycelia were frozen with liquid nitrogen for RNA extraction.

### RNA extraction

RNA was extracted from the in vitro samples with the SV Total RNA Isolation kit (Promega, Madison,WI, USA). Purity of the extracted RNA was assayed with an ND-1000 spectrophotometer (NanoDrop Technologies Inc., Wilmington, DE, USA), and the extracts were stored at −80 °C pending further analysis.

Total RNA of apple fruits was extracted according to Yang et al. [59], with minor changes: aliquots were taken from pooled samples from the leading edges of the six inoculation areas of each apple. The samples were ground to a fine powder in liquid nitrogen and transferred into 50-mL centrifuge tubes with 10 mL of CTAB RNA extraction buffer comprising 100 mM Tris-borate pH 8, 2 M NaCl, 25 mM ethylenediaminetetraacetic acid (EDTA) pH 8, 2 % (w/v) CTAB, 2 % (w/v) polyvinylpolypyrrolidone, and 2 % (v/v) β-mercaptoethanol. The mixture was shaken for 3 min and then incubated at 65 °C for 15 min. Samples were extracted twice with an equal volume of 24:1 (v:v) chloroform:isoamyl alcohol, and the phases were separated by centrifugation at 10,000 × *g* for 10 min. Following centrifugation, LiCl was added to a final concentration of 2.5 M and RNA was allowed to precipitate overnight at 4 °C. The precipitated RNA was pelleted at 4 °C for 30 min at 10,000 × *g*, washed with 70 % ethanol, and resuspended at 65 °C for 3 min in SSTE buffer comprising 10 mM Tris pH 8, 1 M NaCl, 1 mM EDTA pH 8, and 0.5 % (w/v) SDS. Samples were extracted with equal volumes of 24:1 (v:v) chloroform:isoamyl alcohol, and with equal volumes of 24:1:25 (v:v:v) chloroform:isoamyl alcohol:water-saturated phenol, and the phases were separated by centrifugation at 10,000 × *g* for 10 min. The RNA was ethanol-precipitated overnight, and resuspended in diethyl-pyrocarbonate-treated water. The RNA was further treated with Turbo DNAse (Ambion, Austin, TX, USA).

### Preparations of libraries

A 500-ng of total RNA from 11 samples was processed with the TruSeq RNA Sample Preparation Kit v2 (RS-122-2001) (Illumina, San Diego, CA, USA). Libraries were evaluated with the Qubit and TapeStation software (Agilent Technologies, Palo Alto, CA, USA), and sequencing libraries were constructed with barcodes to enable multiplexing of pools of the eight in vitro samples-duplicates of pH 4 3 h, pool pH 4, pH 7 3 h and pool pH 7-in one lane. The results showed 14.5–32.1 million single-end 50-bp reads.

Three individual lanes were used for the in vivo samples and yielded 199.5–253.3 million single-end, 100-bp reads from the duplicates of the leading edges of infected apple tissue and 31.7 million single-end 100-bp reads from healthy apple tissue. All samples were sequenced on a HiSeq 2500 The transcriptome of the healthy apple tissue and the leading edge of infected tissue were sequenced with Trueseq protocols at the Genome Center of the Life Sciences and Engineering Faculty, Technion-Israel Inst. Technology, Haifa, Israel.

### Bioinformatic analysis of RNAseq Data

Three libraries with total single-end, 100-nucleotides-long RNA-seq reads were generated from the in vivo samples and eight libraries with 50-nucleotides-long total single-end RNA-seq reads were generated from the in vitro samples. The libraries contained the following sequences: 1) duplicates of *in-vitro P. expansum* mycelia in pH 4 for 3 h with 21,506,394 and 26,786,825 reads, respectively; 2) duplicates of *in-vitro P. expansum* mycelia in pH 7 for 3 h with 16,745,224 and 32,103,824 reads, respectively; 3) duplicates of pooled samples from all time points during in vitro exposure in medium at pH 4, with 19,665,360 and 14,581,483 reads, respectively; (4) duplicates of pooled samples from all time points during in vitro exposure in medium at pH 7, with 17,324,922 and 19,406,152 reads, respectively; (5) duplicates of leading edge samples of inoculated apple tissue with 253,299,784 and 199,419,262 reads; (6) one sample of healthy apple cv. 'Golden Delicious' tissue, with 31,719,018 reads.

The datasets are available at the NCBI Sequence Read Archive (SRA) under accession number SRP071104.

The Bowtie2 softweare [14] was used to align the RNA-seq outputs against the transcriptome of *P. expansum*. The libraries were aligned against the *P. expansum* genome (downloaded from NCBI Accession no. JQFX00000000.1). The apple samples were also aligned against the *Malus* × *domestica*. Whole Genome v1.0 that was downloaded from GDR (Genome Database for Rosaceae) [60]. RSEM software [61] was used for transcript quantification of the RNAseq data and then the edgeR package [62] was used to calculate differentially expressed genes.

The genes of *P. expansum* were annotated by using BLASTx [63] against the non-redundant NCBI protein database, after which their GO term [64] was assigned by combining BLASTx data and interproscan analysis [65] by means of the BLAST2go software pipeline [16]. GO-enrichment was analysed by using Fisher’s Exact Test with multiple testing correction of FDR [15]. Heatmap and clustering of the genes were visualized by using the R software ggplots2 package [66]. We used a threshold of FDR < 0.05 [15] and the criterion that expression level increased or decreased by a factor greater or less than 2, respectively, i.e., greater than or less than +1 or −1, respectively, on a logarithmic (base 2) scale. The normalized expression value was centered and log 2 transformed for visualization purposes with a script taken from trinity pipeline [67]. The CAZY analysis was done with the CAZymes Analysis Toolkit (CAT) [45]. A heat map of Cazymes in each cluster was plotted by using the R package pheatmap [68].

### Ammonia atmosphere analysis

To create an ammonia vapor atmosphere, 250-mL aliquots of 5 N NaOH with 0 or 50 μM of NH_4_Cl were placed in closed containers for 24 h before use, after which, five apple fruits were placed inside each container for 5 days. Concentrations of 0 and 50 μM NH_4_Cl in NaOH solutions resulted in detected ammonia concentrations of 0 and 22 μM, respectively. All presented gene-expression results are relative to 0 μM NH_4_Cl in 5 N NaOH.

### Gene-expression analysis by qRT-PCR

Real-time qPCR was performed with the StepOnePlus System (Applied Biosystems, Grand Island, New York, USA). PCR amplification was performed with 3.4 μL of cDNA template in 10 μL of reaction mixture containing 6.6 μL of the mix from the SYBR Green Amplification Kit (ABgene, Surrey, UK) and 300 nM of primers; Additional file 10: Tables S1 and Additional file 11: Table S2 (see Supporting Information) list the forward and reverse primers for each of the indicated genes. The PCR was carried out as follows: 10 min at 94 °C, and 40 cycles of 94 °C for 10 s, 60 °C for 15 s, and 72 °C for 20 s. The samples were subjected to melting-curve analysis: efficiencies were close to 100 % for all primer pairs, and all products showed the expected sizes of 70 to 100 bp. All of the samples were normalized to *28S* expression levels, and the values were expressed as the change (increasing or decreasing) of levels relative to a calibrator sample. Results were analyzed with the StepOnePlus software v.2.2.2 (Applied Biosystems, Grand Island, New York, USA). Relative quantification was performed by the ∆∆C_T_ method [69]. The ∆C_T_ value was determined by subtracting the C_T_ results for the target gene from those for the endogenous control gene-18S for apple analysis and 28S for *P. expansum* analysis-and normalized against the calibration sample to generate the ∆∆C_T_ values. Each experiment was performed in triplicate, and three different biological experiments were conducted. One representative set of results is presented as mean values of 2^-∆∆CT^ ± SE for each treatment.

### Availability of data and material

All data presented in this manuscript were deposited in the NCBI Sequence Read Archive (SRA) database under accession number SRP071104.

## Additional files

Additional file 1: Figure S1. GO-enriched terms of genes in cluster 1. GO enrichment was calculated by using Fisher's Exact Test with blast2go software [18]. All *Penicillium expansum* genes served as background for the calculations. The percentages of the GO term in all *P. expansum* genes are marked in red, and the percentages of the GO terms in the gene clusters are marked in blue. (JPG 231 kb) Additional file 2: Figure S2. GO-enriched terms of genes in cluster 2. GO enrichment was calculated by using Fisher's Exact Test with blast2go software [18]. All *Penicillium expansum* genes served as background for the calculations. The percentages of the GO term in all *P. expansum* genes are marked in red, and the percentages of the GO terms in the gene clusters are marked in blue. (JPG 159 kb) Additional file 3: Figure S3. GO-enriched terms of genes in cluster 3. GO enrichment was calculated by using Fisher's Exact Test with blast2go software [18]. All *Penicillium expansum* genes served as background for the calculations. The percentages of the GO term in all *P. expansum* genes are marked in red, and the percentages of the GO terms in the gene clusters are marked in blue. (JPG 212 kb) Additional file 4: Figure S6. GO-enriched terms of genes in cluster 6. GO enrichment was calculated by using Fisher's Exact Test with blast2go software [18]. All *Penicillium expansum* genes served as background for the calculations. The percentages of the GO term in all *P. expansum* genes are marked in red, and the percentages of the GO terms in the gene clusters are marked in blue. (JPG 159 kb) Additional file 5: Figure S7. GO-enriched terms of genes in cluster 7. GO enrichment was calculated by using Fisher's Exact Test with blast2go software [18]. All *Penicillium expansum* genes served as background for the calculations. The percentages of the GO term in all *P. expansum* genes are marked in red, and the percentages of the GO terms in the gene clusters are marked in blue. (JPG 164 kb) Additional file 6: Figure S5. GO-enriched terms of genes in cluster 5. GO enrichment was calculated by using Fisher's Exact Test with blast2go software [18]. All *Penicillium expansum* genes served as background for the calculations. The percentages of the GO term in all *P. expansum* genes are marked in red, and the percentages of the GO terms in the gene clusters are marked in blue. (JPG 159 kb) Additional file 7: Figure S8. GO-enriched terms of genes in cluster 8. GO enrichment was calculated by using Fisher's Exact Test with blast2go software [18]. All *Penicillium expansum* genes served as background for the calculations. The percentages of the GO term in all *P. expansum* genes are marked in red, and the percentages of the GO terms in the gene clusters are marked in blue. (JPG 164 kb) Additional file 8: Figure S9. GO-enriched terms of genes in cluster 9. GO enrichment was calculated by using Fisher's Exact Test with blast2go software [18]. All *Penicillium expansum* genes served as background for the calculations. The percentages of the GO term in all *P. expansum* genes are marked in red, and the percentages of the GO terms in the gene clusters are marked in blue. (JPG 196 kb) Additional file 9: Figure S4. GO-enriched terms of genes in cluster 4. GO enrichment was calculated by using Fisher's Exact Test with blast2go software [18]. All *Penicillium expansum* genes served as background for the calculations. The percentages of the GO term in all *P. expansum* genes are marked in red, and the percentages of the GO terms in the gene clusters are marked in blue. (JPG 123 kb) Additional file 10: Table S1. Fungal primers used in this study. (DOCX 13 kb) Additional file 11: Table S2. Apple primers used in this research. (DOCX 15 kb)

## References

[1] Pitt JI, Hocking AD (2009). *Penicillium* and related genera. Fungi and food spoilage.

[2] Jurick WM, Janisiewicz WJ, Saftner RA, Vico I, Gaskins VL, Park E, Forsline PL, Fazio G, Conway WS (2011). Identification of wild apple germplasm (Malus spp.) accessions with resistance to the postharvest decay pathogens Penicillium expansum and Colletotrichum acutatum. Plant Breed.

[3] Prusky D, McEvoy JL, Saftner R, Conway WS, Jones R (2004). Relationship between host acidification and virulence of *Penicillium* spp. on apple and citrus fruit. Phytopathology.

[4] Hadas Y, Goldberg I, Pines O, Prusky D (2007). Involvement of gluconic acid and glucose oxidase in the pathogenicity of *Penicillium expansum* in apples. Phytopathology.

[5] Yao C, Conway WS, Sams CE (1996). Purification and characterization of a polygalacturonase produced by *Penicillium expansum* in apple fruit. Phytopathology.

[6] Sanchez-Torres P, Gonzalez-Candelas L (2003). Isolation and characterization of genes differentially expressed during the interaction between apple fruit and *Penicillium expansum*. Mol Plant Pathol.

[7] Barad S, Horowitz SB, Moskovitch O, Lichter A, Sherman A, Prusky D (2012). A *Penicillium expansum* glucose oxidase-encoding gene, GOX2, Is essential for gluconic acid production and acidification during colonization of deciduous fruit. Mol Plant-Microbe Interact.

[8] Barad S, Espeso EA, Sherman A, Prusky D. Ammonia activates pacC and patulin accumulation in acidic environment during apple colonization by Penicillium expansum. Mol Plant Pathol. 2015. 10.1111/mpp.12327 PMC6638319 26420024

[9] Yang C, Sudderth J, Dang T, Bachoo RG, McDonald JG, DeBerardinis RJ (2009). Glioblastoma cells require glutamate dehydrogenase to survive impairments of glucose metabolism or Akt signaling. Cancer Res.

[10] Bi F, Barad S, Ment D, Luria N, Dubey A, Casado V, et al. Carbon regulation of environmental pH by secreted small molecules that modulate pathogenicity in phytopathogenic fungi. Mol Plant Pathol. 2015. 10.1111/mpp.12355 PMC6638356 26666972

[11] Penalva MA, Lucena-Agell D, Arst HN (2014). Liaison alcaline: Pals entice non-endosomal ESCRTs to the plasma membrane for pH signaling. Curr Opin Microbiol.

[12] Ballester AR, Marcet-Houben M, Levin E, Sela N, Selma-Lázaro C, Carmona L, Wisniewski M, Droby S, González-Candelas L, Gabaldón T (2015). Genome, transcriptome, and functional analyses of *Penicillium expansum* provide new insights into secondary metabolism and pathogenicity. Mol Plant Microbe Interact.

[13] Benson DA, Cavanaugh M, Clark K, Karsch-Mizrachi I, Lipman DJ, Ostell J, Sayers EW (2013). GenBank. Nucleic Acids Res.

[14] Langmead B, Salzberg SL (2012). Fast gapped-read alignment with Bowtie 2. Nat Methods.

[15] Benjamini Y, Hochberg Y (1995). Controlling the false discovery rate - a practical and powerful approach to multiple testing. J R Stat Soc Ser B Methodol.

[16] Conesa A, Götz S, García-Gómez JM, Terol J, Talón M, Robles M (2005). Blast2GO: a universal tool for annotation, visualization and analysis in functional genomics research. Bioinformatics.

[17] Routledge R (2005). Fisher's exact test.

[18] Li B, Zong Y, Du Z, Chen Y, Zhang Z, Qin G, Zhao W, Tian S (2015). Genomic characterization reveals insights into patulin biosynthesis and pathogenicity in *Penicillium* species. Mol Plant-Microbe Interact.

[19] Zong Y, Li B, Tian S (2015). Effects of carbon, nitrogen and ambient pH on patulin production and related gene expression in *Penicillium expansum*. Int J Food Microbiol.

[20] Snini SP, Tannous J, Heuillard P, Bailly S, Lippi Y, Zehraoui E, Barreau C, Oswald IP, Puel O. The patulin is a cultivar dependent aggressiveness factor favoring the colonization of apples by Penicillium expansum. Mol Plant Pathol. 2015. 10.1111/mpp.12338 PMC6638343 26582186

[21] Prusky D, Yakoby N (2003). Pathogenic fungi: leading or led by ambient pH?. Mol Plant Pathol.

[22] Yadav S, Yadav PK, Yadav D, Yadav KDS (2009). Pectin lyase: a review. Process Biochem.

[23] López‐Pérez M, Ballester AR, González‐Candelas L (2015). Identification and functional analysis of Penicillium digitatum genes putatively involved in virulence towards citrus fruit. Mol Plant Pathol.

[24] Naumann TA, Price NP (2012). Truncation of class IV chitinases from Arabidopsis by secreted fungal proteases. Mol Plant Pathol.

[25] Kumar S, Punekar NS (1997). The metabolism of 4-aminobutyrate (GABA) in fungi. Mycol Res.

[26] Mead O, Thynne E, Winterberg B (2013). Characterising the role of GABA and its metabolism in the wheat pathogen Stagonospora nodorum.

[27] Miyara I, Shafran H, Kramer Haimovich H, Rollins J, Sherman A, Prusky D (2008). Multi-factor regulation of pectate lyase secretion by *Colletotrichum gloeosporioides* pathogenic on avocado fruits. Mol Plant Pathol.

[28] Barad S, Horowitz SB, Kobiler I, Sherman A, Prusky D (2014). Accumulation of the mycotoxin patulin in the presence of gluconic acid contributes to pathogenicity of *Penicillium expansum*. Mol Plant-Microbe Interact.

[29] Iida K, Cox-Foster DL, Yang X, Ko WY, Cavener DR (2007). Expansion and evolution of insect GMC oxidoreductases. BMC Evol Biol.

[30] Alkan N, Davydov O, Sagi M, Fluhr R, Prusky D (2009). Ammonium secretion by *Colletotrichum coccodes* activates host NADPH oxidase activity enhancing host cell death and fungal virulence in tomato fruits. Mol Plant-Microbe Interact.

[31] Alkan N, Meng X, Friedlander G, Reuveni E, Sukno S, Sherman A, Thon M, Fluhr R, Prusky D (2013). Global aspects of pacC regulation of pathogenicity genes in *Colletotrichum gloeosporioides* as revealed by transcriptome analysis. Mol Plant Microbe Interact.

[32] Song X, She X, Yue M, Liu Y, Wang Y, Zhu X, Huang A (2014). Involvement of copper amine oxidase (CuAO)-dependent hydrogen peroxide synthesis in ethylene-induced stomatal closure in Vicia faba. Russ J Plant Physiol.

[33] Cona A, Rea G, Angelini R, Federico R, Tavladoraki P (2006). Functions of amine oxidases in plant development and defence. Trends Plant Sci.

[34] Rea G, Metoui O, Infantino A, Federico R, Angelini R (2002). Copper amine oxidase expression in defense responses to wounding and Ascochyta rabiei invasion. Plant Physiol.

[35] Pongpom P, Cooper CR, Vanittanakom N (2005). Isolation and characterization of a catalase-peroxidase gene from the pathogenic fungus, Penicillium marneffei. Med Mycol.

[36] Miyara I, Shnaiderman C, Meng X, Vargas WA, Diaz-Minguez JM, Sherman A, Thon M, Prusky D (2012). Role of nitrogen-metabolism genes expressed during pathogenicity of the alkalinizing *Colletotrichum gloeosporioides* and their differential expression in acidifying pathogens. Mol Plant-Microbe Interact.

[37] Hammond-Kosack KE, Parker JE (2003). Deciphering plant–pathogen communication: fresh perspectives for molecular resistance breeding. Curr Opin Biotechnol.

[38] Chalutz E, Lieberman M (1977). Methionine-induced Ethylene Production by Penicillium digitatum. Plant Physiol.

[39] Jia YJ, Kakuta Y, Sugawara M, Igarashi T, Oki N, KisAKi M, Shoji T, Kanetuna Y, Horita T, Matsui H (1999). Synthesis and degradation of 1-aminocyclopropane-1-carboxylic acid by Penicillium citrinum. Biosci Biotechnol Biochem.

[40] Eliasson E, Mkrtchian S, Ingelman-Sundberg M (1992). Hormone-and substrate-regulated intracellular degradation of cytochrome P450 (2E1) involving MgATP-activated rapid proteolysis in the endoplasmic reticulum membranes. J Biol Chem.

[41] Lombard V, Golaconda Ramulu H, Drula E, Coutinho PM, Henrissat B (2014). The carbohydrate-active enzymes database (CAZy) in 2013. Nucleic Acids Res.

[42] Zhao Z, Liu H, Wang C, Xu JR (2013). Comparative analysis of fungal genomes reveals different plant cell wall degrading capacity in fungi. BMC Genomics.

[43] Marcet-Houben M, Ballester AR, de la Fuente B, Harries E, Marcos JF, González-Candelas L, Gabaldón T (2012). Genome sequence of the necrotrophic fungus Penicillium digitatum, the main postharvest pathogen of citrus. BMC Genomics.

[44] Cantarel BL, Coutinho PM, Rancurel C, Bernard T, Lombard V, Henrissat B (2009). The Carbohydrate-Active EnZymes database (CAZy): an expert resource for Glycogenomics. Nucleic Acids Res.

[45] Park BH, Karpinets TV, Syed MH, Leuze MR, Uberbacher EC (2010). CAZymes Analysis Toolkit (CAT): web service for searching and analyzing carbohydrate-active enzymes in a newly sequenced organism using CAZy database. Glycobiology.

[46] O'Connell RJ, Thon MR, Hacquard S, Amyotte SG, Kleemann J, Torres MF, Damm U, Buiate EA, Epstein L, Alkan N (2012). Lifestyle transitions in plant pathogenic *Colletotrichum* fungi deciphered by genome and transcriptome analyses. Nature Gen.

[47] Joung JG, Corbett AM, Fellman SM, Tieman DM, Klee HJ, Giovannoni JJ, Fei Z (2009). Plant MetGenMAP: an integrative analysis system for plant systems biology. Plant Physiol.

[48] Creelman RA, Mullet JE (1997). Biosynthesis and action of jasmonates in plants. Annu Rev Plant Biol.

[49] Schaller F (2001). Enzymes of the biosynthesis of octadecanoid-derived signalling molecules. J Exp Bot.

[50] Droby S, Porat R, Cohen L, Weiss B, Shapiro B, Philosoph-Hadas S, Meir S (1999). Suppressing green mold decay in grapefruit with postharvest jasmonate application. J Am Soc Hortic Sci.

[51] Rose USR, Manukian A, Heath RR, Tumlinson JH (1996). Volatile semiochemicals released from undamaged cotton leaves (a systemic response of living plants to caterpillar damage). Plant Physiol.

[52] Ryu CM, Farag MA, Hu CH, Reddy MS, Kloepper JW, Paré PW (2004). Bacterial volatiles induce systemic resistance in Arabidopsis. Plant Physiol.

[53] Falcone Ferreyra ML, Rius SP, Casati P (2012). Flavonoids: biosynthesis, biological functions, and biotechnological applications. Front Plant Sci.

[54] Bouche N, Fromm H (2004). GABA in plants: just a metabolite?. Trends Plant Sci.

[55] Oliver RP, Solomon PS (2004). Does the oxidative stress used by plants for defence provide a source of nutrients for pathogenic fungi?. Trends Plant Sci.

[56] Solomon PS, Oliver RP (2001). The nitrogen content of the tomato leaf apoplast increases during infection by Cladosporium fulvum. Planta.

[57] Ferreira P, Hernandez-Ortega A, Herguedas B, Martinez AT, Medina M (2009). Aryl-alcohol oxidase involved in lignin degradation: a mechanistic study based on steady and pre-steady state kinetics and primary and solvent isotope effects with two alcohol substrates. J Biol Chem.

[58] Hallberg BM, Henriksson G, Pettersson G, Divne C (2002). Crystal structure of the flavoprotein domain of the extracellular flavocytochrome cellobiose dehydrogenase. J Mol Biol.

[59] Yang G, Zhou R, Tang T, Shi S (2008). Simple and efficient isolation of high-quality total RNA from Hibiscus tiliaceus, a mangrove associate and its relatives. Prep Biochem Biotechnol.

[60] Jung S, Staton M, Lee T, Blenda A, Svancara R, Abbott A, Main D (2008). GDR (Genome Database for Rosaceae): integrated web-database for Rosaceae genomics and genetics data. Nucleic Acids Res.

[61] Li B, Dewey CN (2011). RSEM: accurate transcript quantification from RNA-Seq data with or without a reference genome. BMC Bioinformatics.

[62] Robinson MD, McCarthy DJ, Smyth GK (2010). edgeR: a Bioconductor package for differential expression analysis of digital gene expression data. Bioinformatics.

[63] Altschul SF, Gish W, Miller W, Myers EW, Lipman DJ (1990). Basic local alignment search tool. J Mol Biol.

[64] Consortium GO (2013). Gene Ontology annotations and resources. Nucleic Acids Res.

[65] Hunter S, Apweiler R, Attwood TK, Bairoch A, Bateman A, Binns D, Bork P, Das U, Daugherty L, Duquenne L (2009). InterPro: the integrative protein signature database. Nucleic Acids Res.

[66] Team RDC (2009). R: a language and environment for statistical computing.

[67] Haas BJ, Papanicolaou A, Yassour M, Grabherr M, Blood PD, Bowden J, Couger MB, Eccles D, Li B, Lieber M (2013). De novo transcript sequence reconstruction from RNA-seq using the Trinity platform for reference generation and analysis. Nat Protoc.

[68] Kolde R (2012). Pheatmap: pretty heatmaps.

[69] Livak KJ, Schmittgen TD (2001). Analysis of relative gene expression data using real-time quantitative PCR and the 2− ΔΔCT method. Methods.

[70] Suzuki R, Shimodaira H (2013). Hierarchical clustering with P-values via multiscale bootstrap resampling.

[71] Dahl DB (2006). Model-based clustering for expression data via a Dirichlet process mixture model.

[72] Pavlidis P, Noble WS (2003). Matrix2png: a utility for visualizing matrix data. Bioinformatics.

