# Supplementary figures and images for: Fungal and host transcriptome analysis of pH-regulated genes during colonization of apple fruits by Penicillium expansum
Source: BMC Genomics. 2016 May 4;17:330. doi: 10.1186/s12864-016-2665-7 (PMC4855365; doi:10.1186/s12864-016-2665-7)

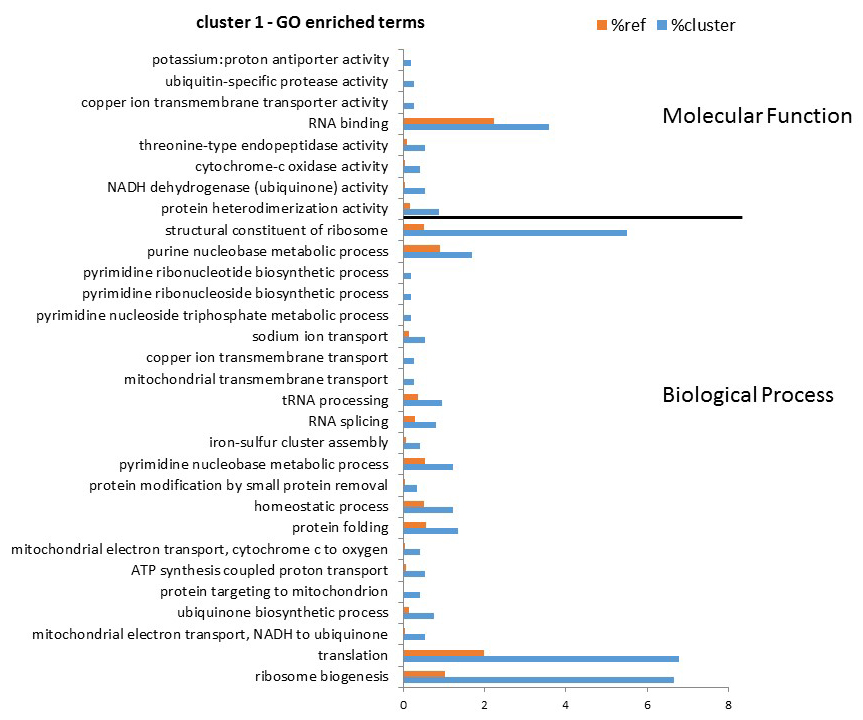

Supplement: Additional file 1: Figure S1. — GO-enriched terms of genes in cluster 1. GO enrichment was calculated by using Fisher's Exact Test with blast2go software [18]. All Penicillium expansum genes served as background for the calculations. The percentages of the GO term in all P. expansum genes are marked in red, and the percentages of the GO terms in the gene clusters are marked in blue. (JPG 231 kb) [file 12864_2016_2665_MOESM1_ESM.jpg]

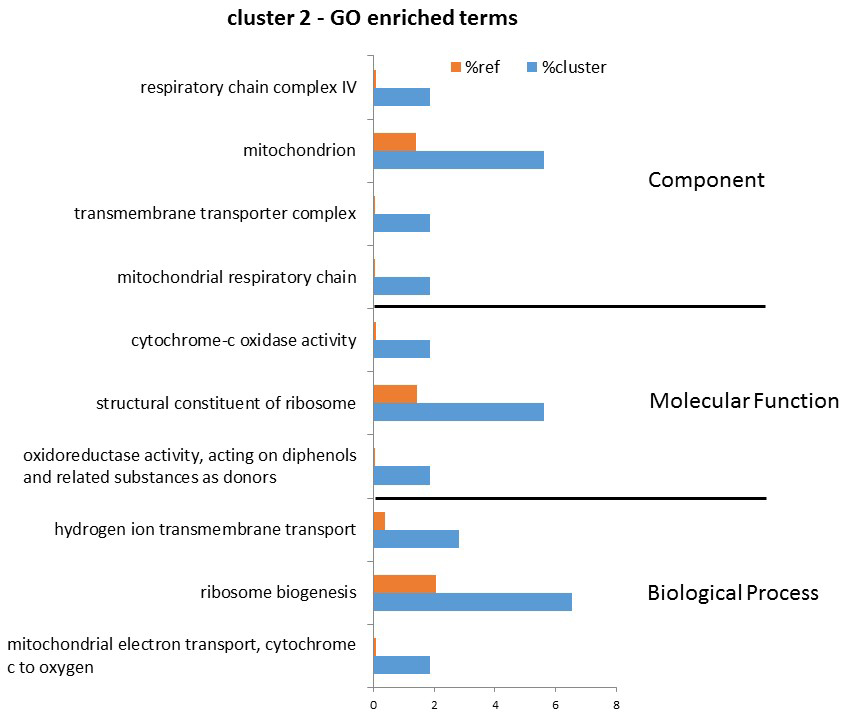

Supplement: Additional file 2: Figure S2. — GO-enriched terms of genes in cluster 2. GO enrichment was calculated by using Fisher's Exact Test with blast2go software [18]. All Penicillium expansum genes served as background for the calculations. The percentages of the GO term in all P. expansum genes are marked in red, and the percentages of the GO terms in the gene clusters are marked in blue. (JPG 159 kb) [file 12864_2016_2665_MOESM2_ESM.jpg]

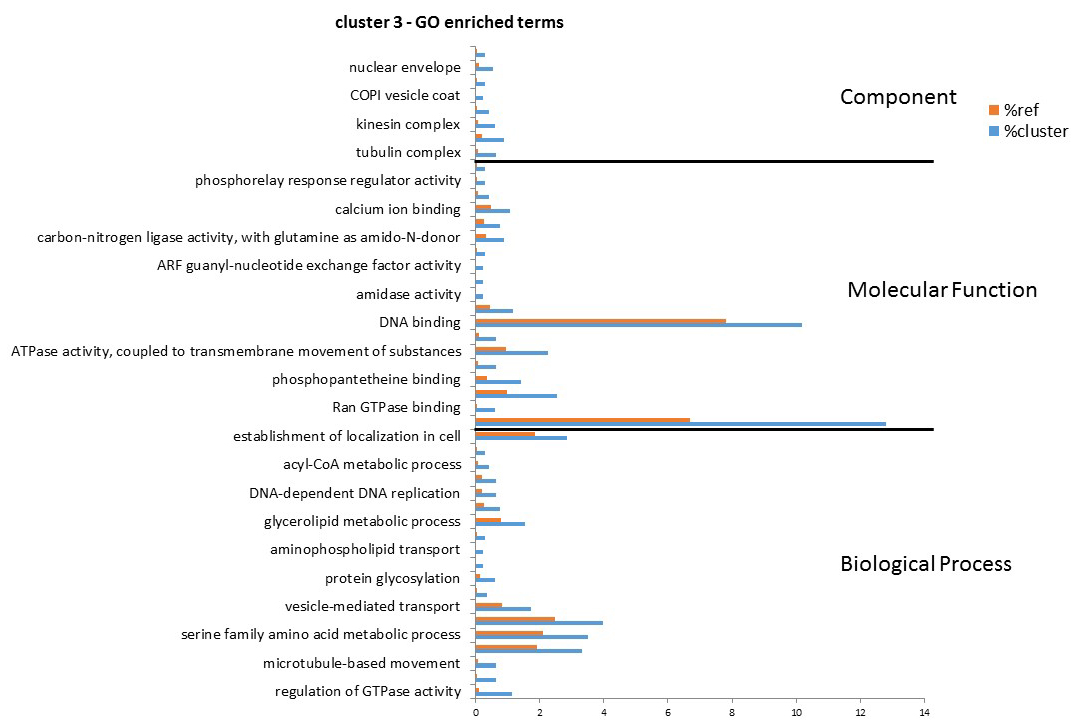

Supplement: Additional file 3: Figure S3. — GO-enriched terms of genes in cluster 3. GO enrichment was calculated by using Fisher's Exact Test with blast2go software [18]. All Penicillium expansum genes served as background for the calculations. The percentages of the GO term in all P. expansum genes are marked in red, and the percentages of the GO terms in the gene clusters are marked in blue. (JPG 212 kb) [file 12864_2016_2665_MOESM3_ESM.jpg]

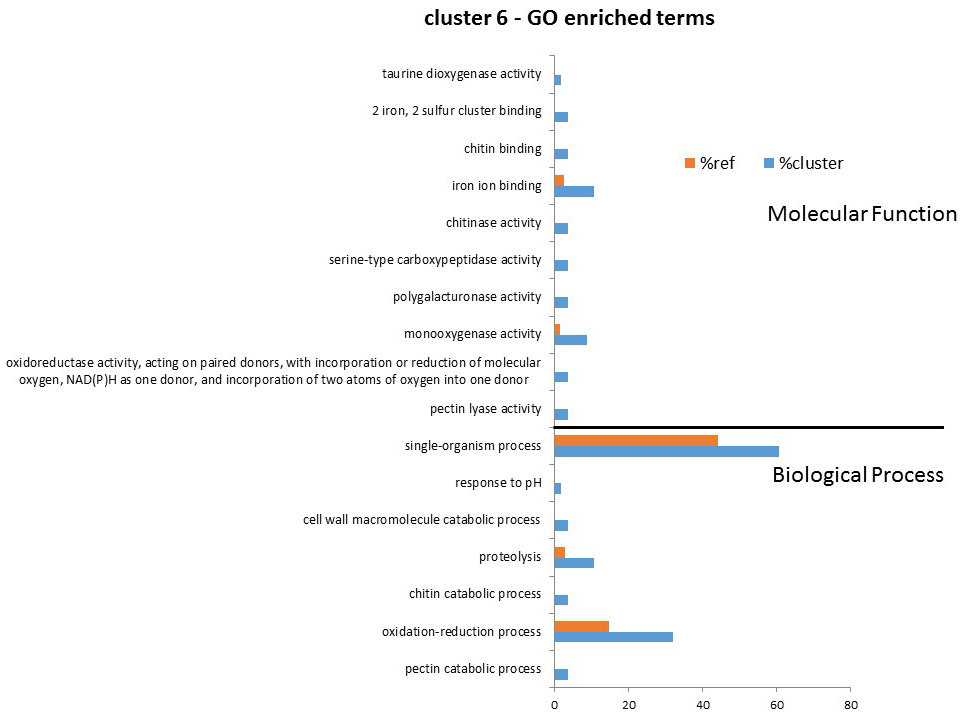

Supplement: Additional file 4: Figure S6. — GO-enriched terms of genes in cluster 6. GO enrichment was calculated by using Fisher's Exact Test with blast2go software [18]. All Penicillium expansum genes served as background for the calculations. The percentages of the GO term in all P. expansum genes are marked in red, and the percentages of the GO terms in the gene clusters are marked in blue. (JPG 159 kb) [file 12864_2016_2665_MOESM4_ESM.jpg]

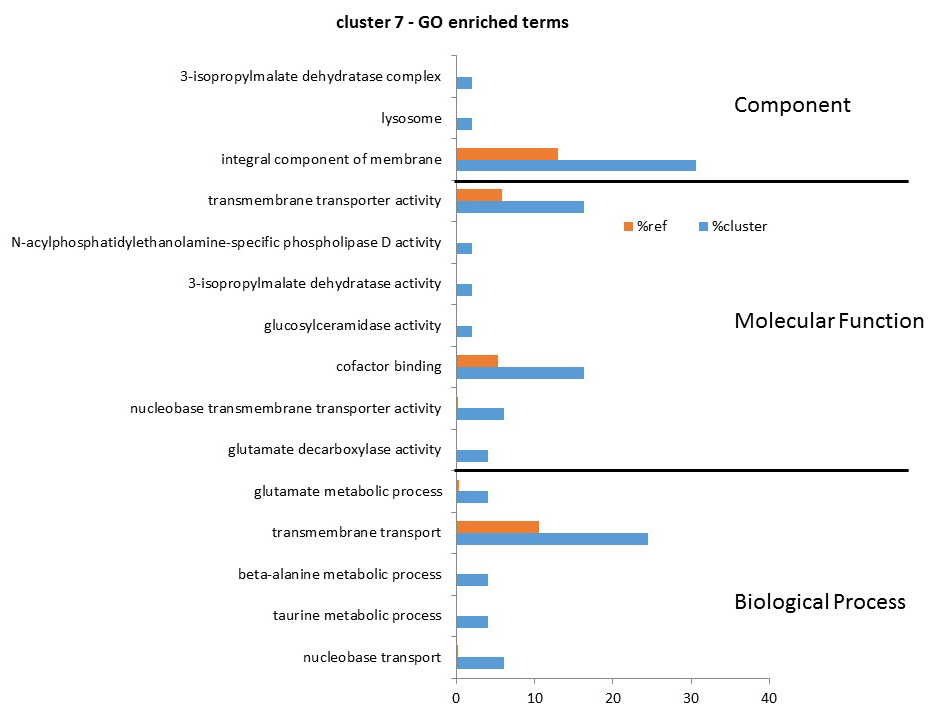

Supplement: Additional file 5: Figure S7. — GO-enriched terms of genes in cluster 7. GO enrichment was calculated by using Fisher's Exact Test with blast2go software [18]. All Penicillium expansum genes served as background for the calculations. The percentages of the GO term in all P. expansum genes are marked in red, and the percentages of the GO terms in the gene clusters are marked in blue. (JPG 164 kb) [file 12864_2016_2665_MOESM5_ESM.jpg]

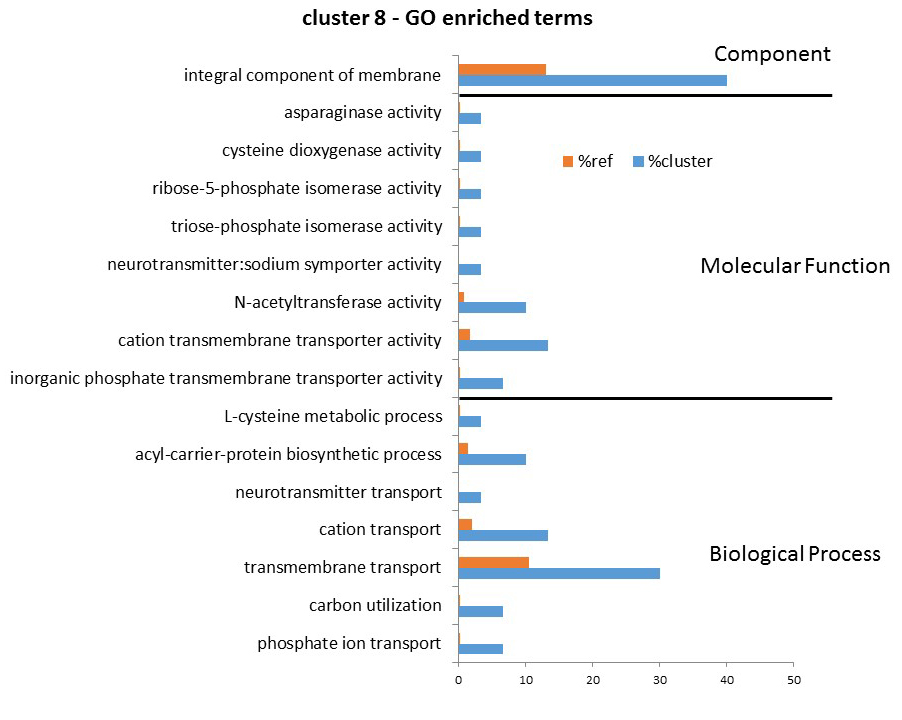

Supplement: Additional file 8: Figure S9. — GO-enriched terms of genes in cluster 9. GO enrichment was calculated by using Fisher's Exact Test with blast2go software [18]. All Penicillium expansum genes served as background for the calculations. The percentages of the GO term in all P. expansum genes are marked in red, and the percentages of the GO terms in the gene clusters are marked in blue. (JPG 196 kb) [file 12864_2016_2665_MOESM8_ESM.jpg]

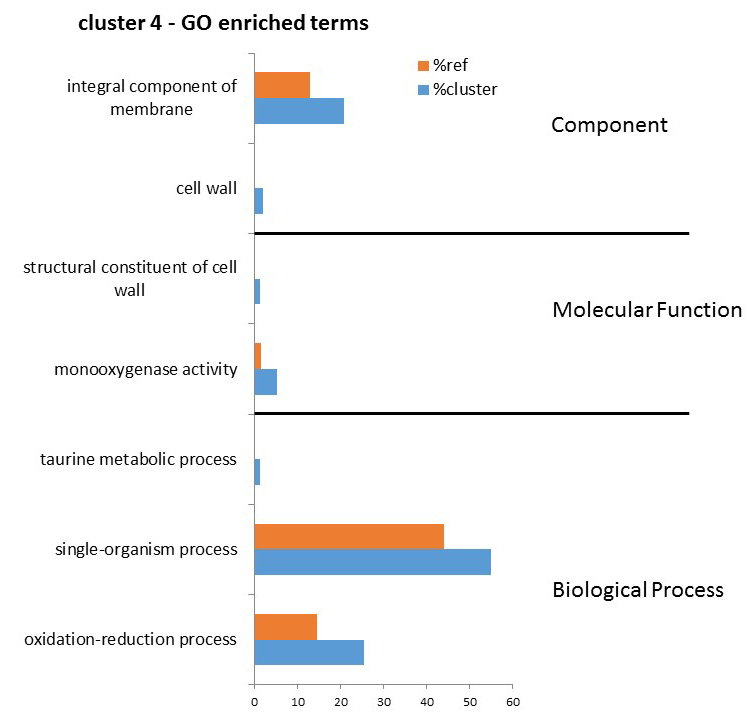

Supplement: Additional file 9: Figure S4. — GO-enriched terms of genes in cluster 4. GO enrichment was calculated by using Fisher's Exact Test with blast2go software [18]. All Penicillium expansum genes served as background for the calculations. The percentages of the GO term in all P. expansum genes are marked in red, and the percentages of the GO terms in the gene clusters are marked in blue. (JPG 123 kb) [file 12864_2016_2665_MOESM9_ESM.jpg]
