# Supplementary material for: Fungal and host transcriptome analysis of pH-regulated genes during colonization of apple fruits by Penicillium expansum
Source: BMC Genomics. 2016 May 4;17:330. doi: 10.1186/s12864-016-2665-7 (PMC4855365; doi:10.1186/s12864-016-2665-7)
Supplement: Additional file 10: Table S1. — Fungal primers used in this study. (DOCX 13 kb) [file 12864_2016_2665_MOESM10_ESM.docx]

**Table S1.** Fungal primers used in this study.

**Fungal primers**

| **Oligonucleotide** | **Sequence 5'→3'** |
| --- | --- |
| F28S | GGAACGGGACGTCATAGAGG |
| R28S | AGAGCTGCATTCCCAAACAAC |
| FPACC1-RT | ATGTATACTATGCCCTGAGCCAA |
| RPACC1-RT | TCGTTCAACGCGTCATATCC |
| Fmep2_Pe-RT | CCCTGCATTGTCTTCATGTT |
| Rmep2_Pe-RT | CAGCGTTGAATCCGAACCA |
| FCuAO_Pe-RT | CACCGATTGGATAATGGGTCTT |
| RCuAO_Pe-RT | GGCAGCTCTTTCGCTGACA |
| FACC_Pe-RT | GACTTGGGATGCGATTGAATATG |
| RACC_Pe-RT | CTGCAAAGCTCTTGCCTTCAT |
| FPelA_Pe-RT | ACCGGTCACTTGCGTGTCA |
| RPelA_Pe-RT | GTGCCGAAACGGAAGGAA |
| FPG_Pe-RT | GGGTTCCAACAGCAAGACCA |
| RPG_Pe-RT | ACCCGGTGATAGTGGAGGATC |
| FGox2_Pe-RT | TTCACAACCGCACCGAGA |
| RGox2_Pe-RT | GGGCGTGTCCAGGAACC |
