# Supplementary material for: Fungal and host transcriptome analysis of pH-regulated genes during colonization of apple fruits by Penicillium expansum
Source: BMC Genomics. 2016 May 4;17:330. doi: 10.1186/s12864-016-2665-7 (PMC4855365; doi:10.1186/s12864-016-2665-7)
Supplement: Additional file 11: Table S2. — Apple primers used in this research. (DOCX 15 kb) [file 12864_2016_2665_MOESM11_ESM.docx]

**Table S2**. Apple primers used in this research.

| **Primers** | **Apple down-regulated genes** |
| --- | --- |
| F: GTTACTTTTAGGACTCCGCC  R: TTCCTTTAAGTTTCAGCCTTG | *Malus domestica* 18S |
| F: CCTGGCAAGGGCTGTAATTG  R: TGCTATTTGGTGGCAACTTGTT | Programmed cell death protein 4-like |
| F: GGTTCTGCTTGAAAAGCTTTGG  R: CCGGCTTCCTAAGCATGTCA | Auxin-repressed kda isoform x1 |
| F: GGTGGACCATCTCACCAAAGA  R: ATGGCACTGTAGCTGGTCAGAA | ap2-like ethylene-responsive transcription factor at2g41710 isoform x2 |
| F: CTGGCTGTGATGTGGAGATTGA  R: TTCCACTCCATAGCCTCCTAAGTT | udp-glucose:glycoprotein glucosyltransferase |
| F: CAGAAAGAAGGAGCCGCAAT  R: ATGCAAAGCGGTTCGATCAT | Senescence-associated carboxylesterase 101-like |
| F: AGGATGCATTCTCTGGTGGAA  R: AAGCTCTGGCTGAGGACATTG | 1-Aminocyclopropane-1-carboxylate oxidase 1 |
| F: AGAGTGGCTAGGGTGCCAGTT  R: AGACCGGTTGAGGTTGAATCG | Anthocyanidin 3-o-glucosyltransferase 5-like |
| F: GAGCTGCGGGTCTTGTTATGA  R: TGGCGGTGACAATGATGCT | Expansin 1 |
| F: GAGCTGACAGAAGTGGAGGAAAC  R: TGCACTTCGTCATCTTCCATTC | ap2-like ethylene-responsive transcription factor at2g41710 isoform x2 |
| F: GTGGCTGGAGATGCTGCAAT  R: CCTTAATATGTCCAACACCCTAT | Lysine-specific histone demethylase 1 homolog 1-like |

| **Primers** | **Apple up-regulated genes** |
| --- | --- |
| F: CAAACACAACCCAAGGCTGTAC  R: AGGTCGGCAAACTCGCAAT | Histone deacetylase hdt3-like |
| F: GCATGAGATGGACCCTCTTCA  R: TCTGTGGGCCTAGCCATTG | Phenylalanine ammonia-lyase 1 |
| F: GTGACACCCACCTTGATAGTCTTG  R: TGGATCCGCACCAATGATTA | Chalcone synthase |
| F: CATAGAGGAATCGACACGAAGAGA  R: AAGAAAGGGCGCAAGTTGAGT | Peroxidase 47 |
| F: TGCTTGTAGGGCTCGGAATC  R: GCCCTTCATCTTCATGTTGTTG | Respiratory burst oxidase homolog protein d-like |
| F: GGTGAGAGCTGTTGTGACTGTGA  R: TGAGGGAATCCAAGTGTTTGG | Lipoxygenase |
| F: TGGAAATGCAGACGTGGATATG  R: CGCTTCTGGGATTCGTAGCA | Indole-3-acetic acid-induced protein arg2 |
| F: TGCGTTGAGCATCGGTCTT  R: ATTTCGCAAACCACCACCTT | Zinc finger an1 domain-containing stress-associated protein 12-like |
| F: ATACTGTCTGTTTCCAAGGCCAAA  R: CAAGTGGTTTACAGAGGGTGAAGA | NADPH--cytochrome p450 reductase isoform x2 |
| F: GGTGGACCATCTCACCAAAGA  R: ATGGCACTGTAGCTGGTCAGAA | Ethylene-responsive transcription factor 2-like |
| F: CCTCCATGACCATCTCCACAA  R: CTGCGTCAAGAGCCACTTTG | Serine threonine-protein kinase-like protein ccr4 |
